# Lyve-1 deficiency enhances the hepatic immune microenvironment entailing altered susceptibility to melanoma liver metastasis

**DOI:** 10.1186/s12935-022-02800-x

**Published:** 2022-12-10

**Authors:** Anna Sophia Jauch, Sebastian A. Wohlfeil, Céline Weller, Bianca Dietsch, Verena Häfele, Ana Stojanovic, Maximilian Kittel, Hendrik Nolte, Adelheid Cerwenka, Michael Neumaier, Kai Schledzewski, Carsten Sticht, Philipp-Sebastian Reiners-Koch, Sergij Goerdt, Cyrill Géraud

**Affiliations:** 1 Section of Clinical and Molecular Dermatology, Medical Faculty Mannheim, Heidelberg University, Mannheim, Germany; 2 Department of Dermatology, Venereology, and Allergology, University Medical Center and Medical Faculty Mannheim, Heidelberg University, and Center of Excellence in Dermatology, 68135 Mannheim, Germany; 3 Department of Immunobiochemistry, Mannheim Institute for Innate Immunoscience (MI3), Medical Faculty Mannheim, Heidelberg University, Mannheim, Germany; 4 Institute for Clinical Chemistry, Medical Faculty Mannheim, Heidelberg University, Mannheim, Germany; 5 NGS Core Facility, Medical Faculty Mannheim, Heidelberg University, Mannheim, Germany; 6 European Center for Angioscience, Medical Faculty Mannheim, Heidelberg University, Mannheim, Germany; 7 Max-Planck-Institute for Biology of Ageing, Cologne, Germany; 8 Skin Cancer Unit, German Cancer Research Center (DKFZ), Heidelberg, Germany

**Keywords:** Lyve-1, Liver, Immune microenvironment, Liver homeostasis, Liver metastasis

## Abstract

**Background:**

Hyaluronan receptor LYVE-1 is expressed by liver sinusoidal endothelial cells (LSEC), lymphatic endothelial cells and specialized macrophages. Besides binding to hyaluronan, LYVE-1 can mediate adhesion of leukocytes and cancer cells to endothelial cells. Here, we assessed the impact of LYVE-1 on physiological liver functions and metastasis.

**Methods:**

Mice with deficiency of *Lyve-1* (Lyve-1-KO) were analyzed using histology, immunofluorescence, microarray analysis, plasma proteomics and flow cytometry. Liver metastasis was studied by intrasplenic/intravenous injection of melanoma (B16F10 *luc2*, WT31) or colorectal carcinoma (MC38).

**Results:**

Hepatic architecture, liver size, endothelial differentiation and angiocrine functions were unaltered in Lyve-1-KO. Hyaluronan plasma levels were significantly increased in Lyve-1-KO. Besides, plasma proteomics revealed increased carbonic anhydrase-2 and decreased FXIIIA. Furthermore, gene expression analysis of LSEC indicated regulation of immunological pathways. Therefore, liver metastasis of highly and weakly immunogenic tumors, i.e. melanoma and colorectal carcinoma (CRC), was analyzed. Hepatic metastasis of B16F10 *luc2* and WT31 melanoma cells, but not MC38 CRC cells, was significantly reduced in Lyve-1-KO mice. In vivo retention assays with B16F10 *luc2* cells were unaltered between Lyve-1-KO and control mice. However, in tumor-free Lyve-1-KO livers numbers of hepatic CD4^+^, CD8^+^ and regulatory T cells were increased. In addition, iron deposition was found in F4/80^+^ liver macrophages known to exert pro-inflammatory effects.

**Conclusion:**

Lyve-1 deficiency controlled hepatic metastasis in a tumor cell-specific manner leading to reduced growth of hepatic metastases of melanoma, but not CRC. Anti-tumorigenic effects are likely due to enhancement of the premetastatic hepatic immune microenvironment influencing early liver metastasis formation.

**Supplementary Information:**

The online version contains supplementary material available at 10.1186/s12935-022-02800-x.

## Background

Liver sinusoidal endothelial cells (LSECs) are a prime example of organ-specific endothelial cells (ECs) that form the hepatic vascular niche. Their morphology is unique as they are equipped by fenestrae and lack a basement membrane. Among their functions are the angiocrine instruction of surrounding cells and control of organ functions by surface expression or secretion of multiple factors [1, 2]. Angiocrine secretion of Wnt and Hgf is crucial for the regulation of liver size and liver regeneration [3–5]. Moreover, by the secretion of Bmp2 and Bmp6 LSECs control systemic and hepatic iron homeostasis [6, 7]. Besides, LSECs are an important immunological component as they contribute to tolerance induction [8].

Notably, LSECs are also decisively involved in almost all liver diseases such as liver fibrosis, carcinogenesis or hepatic tumor metastasis [9, 10]. The differentiation of LSECs is critically controlled by the transcription factor *Gata4*. Loss of *Gata4* in LSECs drives sinusoidal capillarization leading to anemia in the fetal liver due to disturbed hepatic hematopoiesis [11]. In the adult liver its loss in LSECs results in a profibrotic angiocrine switch mediating perisinusoidal fibrosis and hepatopathy [12]. Besides, activation of hepatic endothelial Notch signaling also changes endothelial differentiation towards a continuous EC phenotype [13]. LSECs represent the first cells circulating tumor cells encounter when disseminating to the liver. E-selectin, ICAM1 or Clec4g mediate tumor cell adhesion and thus facilitate liver metastasis [14–16]. An influence of Notch signaling on disease pathology is highlighted as upregulation in hepatic ECs protects from liver metastasis of colorectal carcinoma and cutaneous melanoma, via downregulation of ICAM1 [13]. Besides, deposits of fibronectin alongside the hepatic endothelium were shown to bind circulating tumor cells [17]. Moreover, LSECs were shown to critically control liver metastasis by the secretion of inflammatory cytokines, such as Il-1, Mif or Cxcl12 [18, 19].

By expression of a variety of surface receptors, such as Stabilin-1 (Stab1), Stabilin-2 (Stab2), LYVE-1 or CD32b, LSECs contribute to the clearance of circulating factors from the blood [20]. In this regard, the physiologic involvement of LYVE-1/Lyve-1 in blood clearance has not yet been fully defined, although it is known to mediate endocytosis of hyaluronan. LYVE-1/Lyve-1 is expressed on subsets of endothelial cells, mainly the lymphatic endothelium [21] and sinusoidal vascular ECs such as LSECs [22]. In comparison to the human system, LYVE-1/Lyve-1 expression appears to be broader on vascular ECs in mice, as it is widely expressed during embryonic development and is also found on subsets of vascular EC in the adult heart, spleen, adrenal gland and lungs [23, 24]. Besides ECs, subtypes of macrophages, such as some tumor-infiltrating macrophages, also show expression of LYVE-1/Lyve-1 [25, 26]. However, hepatic Kupffer cells have been described to be Lyve-1 negative [25]. The glycoprotein LYVE-1/Lyve-1 can bind hyaluronan, an extracellular matrix glycosaminoglycan, and functions as an endocytic receptor [27] similar to CD44 [21]. Recently, interaction of Lyve-1 with hyaluronan was found to be involved in lymphatic trafficking of dendritic cells [28]. Regarding cancer metastasis, in vitro studies imply LYVE-1 to be involved in tumor cell adhesion [29]. This can be taken advantage of therapeutically, as administration of a monoclonal anti-Lyve-1 antibody strongly decreased tumor formation and lymphatic metastasis of breast cancer in a mouse model [30]. As of yet, the involvement of LYVE-1/Lyve-1 in tumor cell adhesion to LSECs and hepatic metastasis has not been studied.

Knockout mouse models with varying targeting strategies of the *Lyve-1* locus were generated to decipher the function of Lyve-1. In general, the loss of Lyve-1 does not result in reduced viability or fertility or obvious pathological organ dysfunction. Huang et al. described distended luminal size of lymphatic vessels in the livers of Lyve-1 deficient mice and hypothesized that hyaluronan or PDGF-BB, both ligands of Lyve-1, may act as opening sensors for lymphatic vessels [31]. However, in the study of Gale et al. lymphatic fluid drainage, hyaluronan plasma levels, organ development, leukocyte distribution and subcutaneous tumor growth were unchanged [32]. Therefore, they hypothesized that other hyaluronan receptors, such as CD44, may compensate for the loss of Lyve-1 in knockout mice. However, Lyve-1 and CD44 double knockout mice also show no obvious pathological lymphatic phenotype, but a tendency towards increased numbers of leukocytes in the peritoneal cavity after inflammatory stimulation [33]. Yet, the role of Lyve-1 in hepatic endothelial differentiation and function including metabolic zonation and pathological processes in the liver, such as hepatic metastasis, has not been studied in detail.

Therefore, we here address the role of Lyve-1 in these processes. In this regard, hepatic endothelial differentiation, angiocrine functions and involvement in disease pathogenesis were studied in mice with a constitutive knockout of Lyve-1 (Lyve-1-KO) [31]. Hepatic metastasis of both CRC and CM were studied by splenic or intravenous injections in Lyve-1-KO. We show that Lyve-1 influences tumor-specific hepatic metastasis of melanoma but not CRC without interfering with angiocrine organ functions of LSECs.

## Methods

### Animal ethics

The animal ethics Committee of Baden-Wuerttemberg (Regierungspräsidium Karlsruhe) approved all animal experiments. In adherence to the Guide for the Care and Use of Laboratory Animals published by the National Academy of Sciences the animal care was performed.

### Animal characterization and experiments

Mice between 6 to 18 weeks of age were used for animal characterization and routine analysis. B6.129S1-*Lyve1*^*tm1Lhua*^/J (JAX stock #006221) mice with C57BL/6 background were utilized as Lyve-1^−/−^ group [31]. Littermates bred in a heterozygous mating mode and purchased wild-type C57BL/6 J mice (Janvier Labs, Le Genest-Saint-Isle, France) served as control group (Ctrl). Heterozygous mice were not used. For DNA extraction and genotyping KAPA HotStart Mouse Genotyping Kit (KK7352, Merck, Darmstadt, Germany) and primers (Metabion international AG, Planegg/Steinkirchen, Germany) were utilized (Additional file 3: Table S1). With a 12 h/12 h day/night cycle all mice were hosted in single ventilated cages (Sealsafe plus DGM™, Tecniplast, Buguggiate, Italy) under specific-pathogen free conditions. Mice were fed ad libitum with a standard rodent diet (V1534-000, Ssniff, Soest, Germany) and always had free access to water.

### Cell lines

B16F10 *luc2* mouse melanoma cells, WT31 mouse melanoma cells and MC38 colorectal cancer cells were used for in vivo experiments. B16F10 *luc2* cells were bought from ATCC (Manassas, VA, USA). WT31 mouse melanoma cells [34] were kindly provided by O. Sansom (Beatson Institute for Cancer Research, Glasgow, Scotland). The MC38 colorectal cancer cells were a gift from S. Herzig (Helmholtz Zentrum Munich, Germany).

The same passage of the respective cell lines was utilized for in vivo experiments. After thawing, B16F10 *luc2* and WT31 cells were held in RPMI Medium (61870044, Gibco™, Thermo Fisher Scientific, Waltham, MA, USA) and MC38 cells were held in DMEM Medium (61965059, Gibco™, Thermo Fisher Scientific, Waltham, MA, USA) respectively with 10% fetal bovine serum (10270106, Gibco™, Thermo Fisher Scientific, Waltham, MA, USA) and 1% of penicillin/streptomycin (15140122, Gibco™, Thermo Fisher Scientific, Waltham, MA, USA) at 37 °C in air with 5% CO_2_. After thawing of the cells, they were prepared for the in vivo experiments within maximum 1 week. Thereby, the cells were passaged not more than three times. The cells used for the in vivo experiments were mycoplasma-free tested by PCR. Cell authentication was confirmed by cell morphology and pigmentation status.

### Liver and lung colonization assay

Liver and lung colonization assays were performed as previously established and described [13, 35]. Female mice starting with an age of 11 weeks were used in vivo experiments. All experiments were performed with age matched mice.

To study liver colonization, spleen injections or intravenous injections of tumor cells were performed. After spleen injection of 1.5 × 10^5^ MC38 colorectal carcinoma cells, the mice were sacrificed at day 21. When 1.5 × 10^5^ B16F10 *luc2* cells were injected into the spleen, the mice were sacrificed after 14 days and BLI measurement of the liver was performed. 1.25 × 10^6^ WT31 melanoma cells were applied by tail vein injection and mice were sacrificed after 19 days. When WT31 melanoma cells were injected into the spleen, 0.2 × 10^5^ cells were used and mice were analyzed at day 21. To analyze the retention of melanoma cells in the liver 3 × 10^5^ B16F10 *luc2* cells were injected into the spleen and analysis was performed 90 min after intrasplenic injection by BLI.

### BLI

For bioluminescence imaging (BLI) an IVIS® Lumina LT In Vivo Imaging System (Caliper Life Sciences, Perkin Elmer, Waltham, MA, USA) was used. Ten minutes after intraperitoneal application of luciferin (D-luciferin 1-(4,5-dimethoxy-2-nitrophenyl)ethyl ester, 7903, 30 mg/ml, BioVision, Milpitas, CA, USA), mice were sacrificed, livers were removed, put into a Petri dish (664160, Greiner Bio One, Kremsmünster, Austria) and were imaged ex vivo with the IVIS® Lumina LT with an exposure time of 45 s.

### Iron quantification in liver

The iron in mouse liver lysates was measured with the Iron Assay KIT (MAK025-1KT, Merck, Darmstadt, Germany) according to the manufacturer’s protocol.

### LSEC isolation

LSEC isolation was performed as previously described [36]. Livers of Ctrl (C57BL/6J) and B6.129S1-*Lyve1*^*tm1Lhua*^/J with C57BL/6 background [31] were perfused with a 0.05% collagenase containing amino acid/ saccharide calcium-deprived medium (C2674, Sigma–Aldrich, St. Louis, MO, USA) via the portal vein. Mice were sacrificed and livers were excised. Livers were minced mechanically and digested at 38 °C in a collagenase/Gey’s balanced salt solution (G9779, Sigma-Aldrich, St. Louis, MO, USA). Afterwards livers were filtered through a 250 µm mesh. A gradient was applied by 35% Nycodenz (1002424, Axis-Shield, Alere Technologies, Oslo, Norway) and cells were separated. Utilizing anti-CD146 MicroBeads (ME-9F1, 130-092-007, Miltenyi Biotech, Bergisch Gladbach, Germany) LSECs were isolated by magnetic-activated cell sorting according to the manufacturers’ instructions. Purity of LSECs was checked by their positivity for CD31 and/or Stab2 by flow cytometry with a BD FACSCanto II (BD Biosciences, Franklin Lakes, NJ, USA). For additional analysis, LSECs were pooled from three mice.

### Microarray data^33^

RNA isolation and preparation for microarray analysis were performed as previously described [36]. Utilizing arrays MoGene-2_0-st from Affymetrix (Santa Clara, CA, USA) the gene expression profiling was executed. cDNA and cRNA were synthetized and hybridized to the arrays in accordance with manufacturer’s recommendation. For annotating the arrays a custom CDF Version 18 (MoGene-2_0-st) with Entrez based gene definitions was utilized. Normalizing raw fluorescence intensity values was performed according to quantile normalization. By using a commercial software package SAS JMP7 Genomics, version4, from SAS (SAS Institute, Cary, NC, USA) differential gene expression was analyzed with the One-Way-ANOVA. Level of significance was determined as a false positive rate of a = 0.05 with FDR correction. The raw as well as normalized data are accessible in the Gene Expression Omnibus database (GEO) (http://www.ncbi.nlm.nih.gov/). The GEO accession number is GSE199055.

### Flow cytometric analysis of liver immune cells

Single cell suspensions from liver tissue were first incubated with Zombie Aqua™ fluorescent dye (Biolegend) to label dead cells. Following Fc-receptor blockade with a CD16/32-producing hybridoma supernatant, cells were incubated with antibodies targeting surface molecules. Cells were fixed and permeabilized using eBioscience™ Intracellular Fixation & Permeabilization reagents (Invitrogen™) according to manufacturer’s instructions. Intracellular staining was performed in permeabilization buffer. All antibodies were purchased from Biolegend and BD Biosciences. Data were acquired by LSRFortessa™ X-20 Flow Cytometer (BD) and analyzed using FlowJo™ Software (BD).

### Statistical analysis

Prism 8 (GraphPad Software, La Jolla, CA, USA) was used for performing statistical analysis. Unpaired, two-tailed t-test or Mann–Whitney *U*-test were applied for statistical testing depending on whether normality was proven by a Shapiro–Wilk test or not. If not stated differently in the figure legends, an unpaired, two-tailed t-test was used. For the analysis of the number of hepatic metastases after spleen injection of WT31 melanoma two outliers were identified by the ROUT method in the Lyve-1-KO group and excluded for the plot and analysis. A *P*-value < 0.05 was regarded as statistically significant. The graphs represent the mean ± SEM.

For a detailed description of analysis of blood parameters, plasma proteomics, histological methods, antibodies, image acquisition and immunoblot please refer to the Additional file 2.

## Results

### Analysis of the hepatic morphology and architecture.

**Fig. 1:**
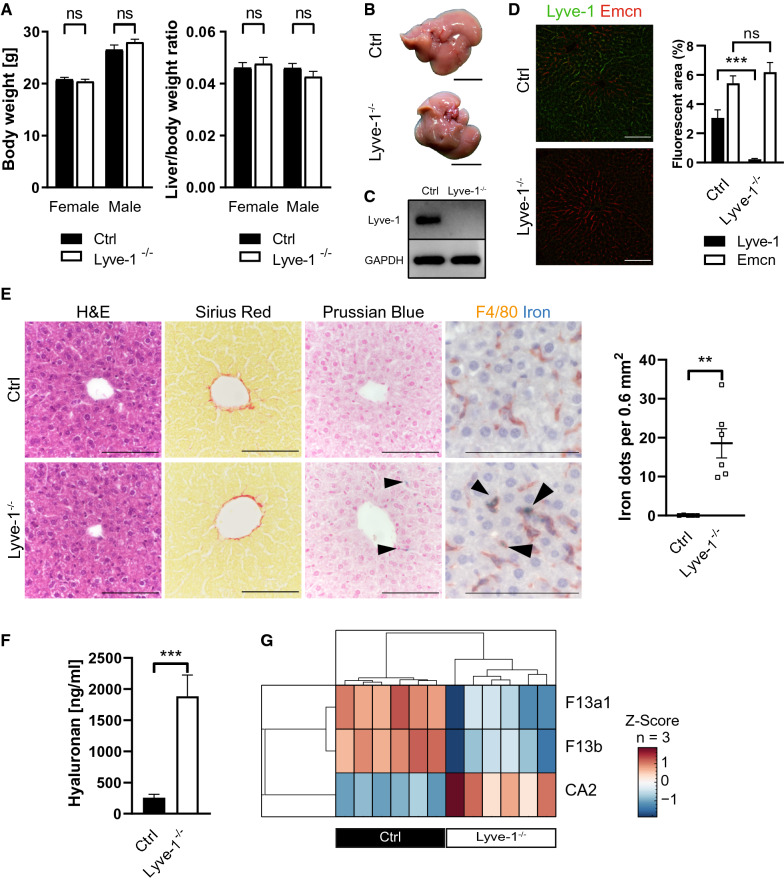
Phenotypical and histological characterization of Lyve-1^−/−^ livers. Iron deposition in F4/80^+^ cells in Lyve-1^−/−^ livers. Increased hyaluronan and carbonic anhydrase 2 levels as well as decreased levels of factor 13a1 and factor 13b in plasma of Lyve-1^−/−^ mice. **A** Body weight (female, *P* = 0.3912, n = 12/group; male, *P* = 0.1662, n = 11/group) and liver/body weight ratio of Ctrl and Lyve-1^−/−^ mice (female, *P* = 0.2343, Mann-Whitney *U*-test, n = 6-7/group; male, *P* = 0.2225, n = 6/group). **B** Representative pictures of Ctrl and Lyve-1^−/−^ livers (female, n = 6/group). Scale bars: 1 cm. **C** Immunoblot of Lyve-1 in Ctrl and Lyve-1^−/−^ livers (female and male, n = 4/group). **D** Immunofluorescence stainings for Lyve-1 and Emcn in Ctrl and Lyve-1^−/−^ liver tissue (female, n = 6/group) and quantification of Lyve-1 (*P* = 0.005) and Emcn (*P* = 0.3905) in Ctrl and Lyve-1^−/−^ liver tissue (female, n = 6/group). Scale bars: 100 µm. **E** H&E and Sirius red stainings of Ctrl and Lyve-1^−/−^ livers (female, n = 5/group). Prussian blue stainings and immunohistochemistries for F4/80 with Prussian blue co-staining of Ctrl and Lyve-1^−/−^ livers (female, n = 6/group). Iron deposits quantified in Prussian blue stained Ctrl and Lyve-1^−/−^ liver slides (female, *P* = 0.0043, Mann-Whitney *U*-test, n = 5-6/group). Scale bars: 100 µm. Black arrows highlight iron deposition. **F** Hyaluronan concentration in plasma of Ctrl and Lyve-1^−/−^ mice (female, 12 weeks, *P* = 0.0008, n = 6/group) measured with Hyaluronan DuoSet ELISA (DY3614-05, R&D Systems, Minneapolis, MN, USA). **G** Proteomics of plasma from Lyve-1-KO and Ctrl mice were performed. A clustered heatmap with significantly regulated proteins is presented (q-values: CA2: 0.0153; F13b: 0.018; F13a: 0.028). Data information: * *P* < 0.05; ** P < 0.01; *** *P* < 0.001; **** *P* < 0.0001; *ns *not significant

Mice with a constitutive knockout (KO) of Lyve-1 (Lyve-1-KO) [31] were used to investigate the influence of Lyve-1 on hepatic endothelial cell differentiation and angiocrine functions. Body weight, liver to body weight ratio and body length were unaltered in Lyve-1-KO mice as compared to control (Ctrl) mice (Fig. 1A; Additional file 1: Fig. S1A). Macroscopically, Lyve-1-KO livers did not show gross alterations (Fig. 1B) and standard liver enzymes ALT, AST and GLDH were unaltered (Additional file 1: Fig. S1B). The knockout of *Lyve-1* was confirmed both by western blotting and immunofluorescence stainings of liver tissue (Fig. 1C, D). While Lyve-1 was expressed in periportal and midlobular LSECs in the livers of Ctrl mice only, Emcn was found in pericentral areas of both groups. The expression and quantified area of Emcn was not extended in Lyve-1-KO indicating maintenance of LSEC zonation. On routine histology (H&E, sirius red, PAS) of the liver, no fibrosis, necrosis, overt inflammation or malformations were observed. Surprisingly, prussian blue staining revealed significantly increased iron deposition in Lyve-1-KO livers, which was absent in Ctrl livers (*P* = 0.0043). Co-staining of F4/80 with prussian blue showed that iron deposits were in F4/80^+^ cells in the Lyve-1-KO livers (Fig. 1E). The total iron concentration of liver lysates was similar in both groups (Additional file 1: Fig. S1C). However, the iron concentration was significantly decreased in plasma of Lyve-1-KO mice as compared to Ctrl (Additional file 1: Fig. S1C). Hepcidin, the main hormone regulating iron metabolism and homeostasis, was analyzed in the plasma and was unaltered between both groups (Additional file 1: Fig. S1C).

### Determination of plasma clearance functions of Lyve-1.

As Lyve-1 is described as endocytic receptor of hyaluronan, plasma hyaluronan concentrations were determined. The concentration of hyaluronan was significantly increased in plasma of Lyve1-KO as compared to Ctrl (*P* = 0.0008) (Fig. 1F; Additional file 1: Fig. S1D, E). To assess Lyve-1 functions in plasma clearance and its global impact of plasmatic protein composition, unbiased data-independent mass spectrometry based proteomic analyses of plasma were performed (n = 6 per genotype). In total, 406 protein groups were quantified followed by a two-sided t-test and permutation-based FDR correction. This analysis revealed three significantly different regulated protein groups including a two-fold decreased abundance of Coagulation factor XIII A and B chain (F13b and F13a1) in Lyve-1 deficient mice compared to Ctrl. Additionally, increased levels of carbonic anhydrase 2 (CA2) were detected (Fig. 1G).

Last, platelets were thoroughly analyzed, since Lyve-1 is also expressed on megakaryocytes [37]. Moreover, increased erythrocyte extravasation into the liver with secondary removal by macrophages could be one reason for increased iron deposition in hepatic macrophages. However, platelet counts and function as well as tests of hemostasis did not show significant differences between the two groups Additional file 1: Figure S2A, B) indicating that iron depositions are likely not due to impaired platelet functions or hemostasis.

### Characterization of endothelial marker expression by immunofluorescence stainings and gene expression by microarray gene expression profiling of isolated LSEC

**Fig. 2:**
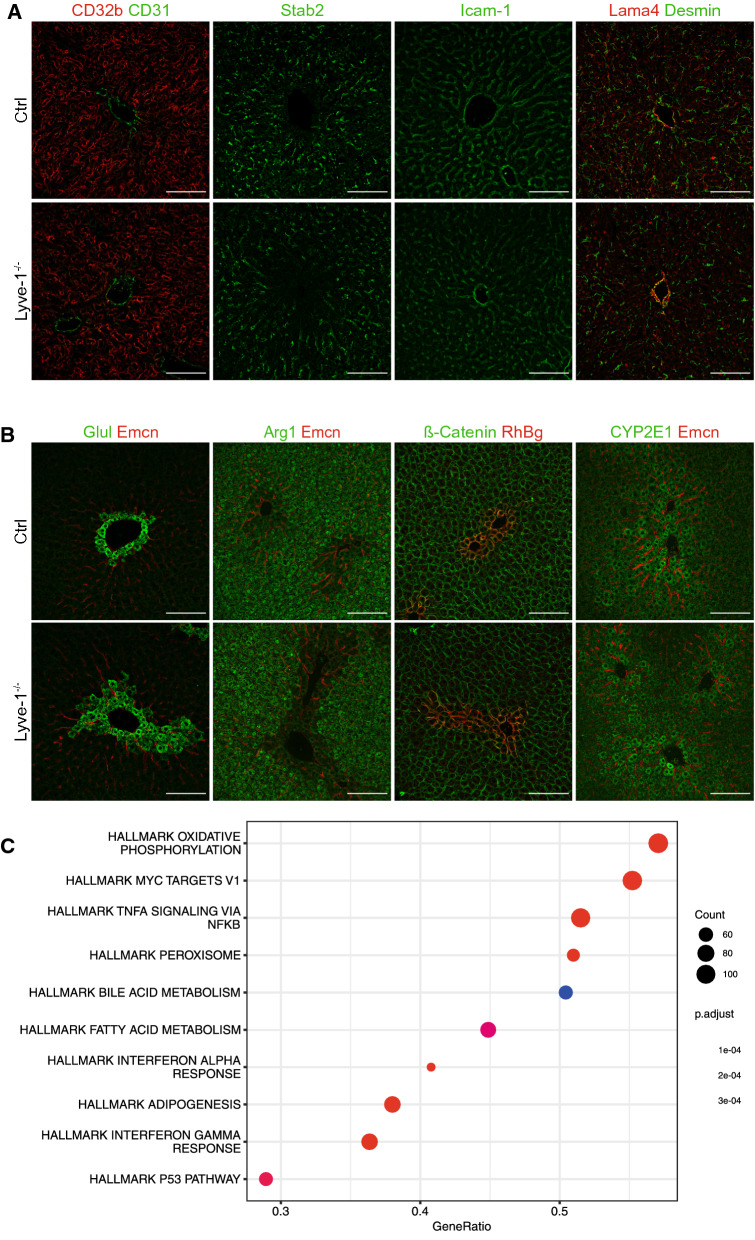
Histological analysis of Ctrl and Lyve-1^−/−^ mice revealed no differences in endothelial differentiation, hepatic metabolic zonation or extracellular matrix deposition.** A** Immunofluorescence stainings of Ctrl and Lyve-1^−/−^ livers for CD32b and CD31, Stab2, Icam-1, Lama4 and Desmin (female, n = 5–6/group). Scale bars: 100 µm. **B** Immunofluorescence stainings of Ctrl and Lyve-1^−/−^ livers show Glul, Emcn, Arg1, β-Catenin, RhBg, CYP2E1 (female, n = 6/group). Scale bars: 100 µm. **C** Affymetrix gene microarray (gene chip) analysis of cDNA from LSECs of Ctrl and Lyve-1^−/−^ mice was performed for gene expression profiling. A dot plot of an overall representation analysis of HALLMARK pathways is presented. The number of regulated genes by count and gene ratio and the adjusted *P* value of corresponding pathways are presented. Data information: * *P* < 0.05; ** *P* < 0.01; *** *P* < 0.001; **** *P* < 0.0001; *ns* not significant

To study the role of Lyve-1 for the sinusoidal phenotype of LSECs, endothelial differentiation was analyzed in detail. Marker proteins of continuous endothelial cells, such as Emcn or CD31, were expressed in pericentral areas or weakly throughout the sinusoids, while markers for sinusoidal endothelial cells, such as CD32b and Stab2, were broadly expressed in sinusoids of Lyve-1-KO and controls. Besides, expression of Icam-1, a pan-endothelial marker and an adhesion molecule, did not differ between both groups (Figs. 1D, 2A). To assess the periendothelial matrix and stellate cell activation Lama4 and Desmin were analyzed. Both were not increased in Lyve-1-KO mice. Capillarization, i.e. formation of a basement membrane, is usually associated with increased deposition of Lama4 and increased numbers of activated Desmin-positive stellate cells. Therefore, these findings demonstrate that the loss of Lyve-1 does not result in capillarization of hepatic sinusoids, i.e. Lyve-1 itself is not a regulator of the sinusoidal phenotype in LSECs. Besides the specialized sinusoidal phenotype, specific angiocrine functions are a hallmark of LSECs. To investigate the role of Lyve-1 in this regard, metabolic zonation that is controlled by angiocrine wnt-signalling was analyzed. Therefore, Glul, Arg1, RhBg and CYP2E1, marker proteins for metabolic liver zonation, were stained. Hepatic metabolic zonation and membranous expression of β-catenin on hepatocytes remained unchanged in Lyve-1-KO livers compared to Ctrl livers (Fig. 2B) indicating functionality of the angiocrine wnt-beta-catenin axis. To comprehensively assess the impact of Lyve-1 deficiency on gene expression in LSECs, microarray gene expression profiling was performed on mRNA from isolated LSECs of Lyve1-KO and Ctrl mice. Even though there was no single gene significantly dysregulated, an overall representation analysis of HALLMARK pathways revealed regulation of Myc targets as well as immunological pathways involved in TNF-α, Interferon-α and -γ signaling (Fig. 2C). Altogether, these data show that knockout of *Lyve-1* did not affect the hallmarks of LSEC-specific endothelial differentiation or angiocrine functions but appeared to affect immunological pathways.

### Influence of Lyve-1 on hepatic metastasis of colorectal carcinoma and melanoma

**Fig. 3:**
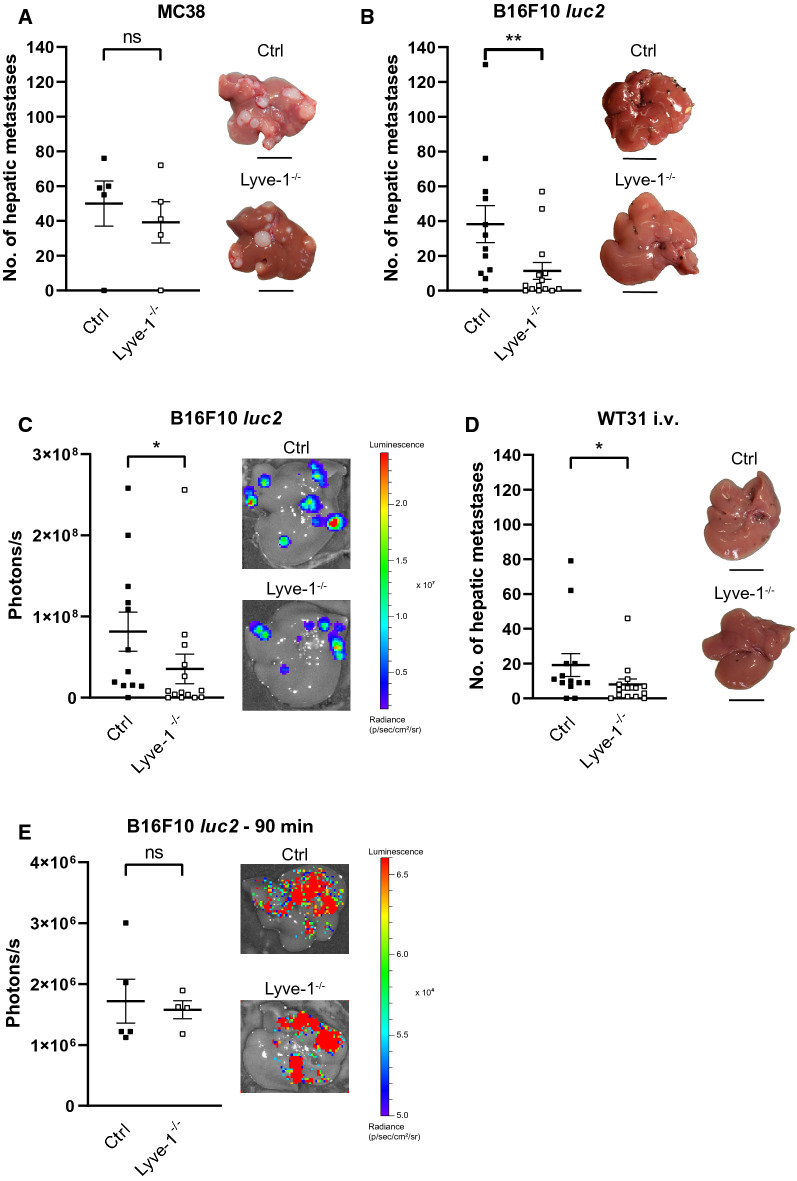
The loss of Lyve-1 protects against hepatic melanoma metastasis.** A** After spleen injection of MC38 colorectal carcinoma cells the numbers of macroscopic visible hepatic metastases (50 vs. 39.2, *P* = 0.5563) were quantified in Ctrl (n = 5) and Lyve-1^−/−^ (n = 5) mice at day 21. Photographs of livers with CRC metastases are shown. Scale bars: 1 cm. **B** Following intrasplenic injection of B16F10 *luc2* melanoma cells, macroscopic visible hepatic metastases were quantified in Ctrl (n = 12) and Lyve-1^−/−^ (n = 14) mice at day 14 (38.25 vs. 11.36, *P* = 0.0093, Mann–Whitney *U*-test). Representative photographs of livers with metastases are shown. Scale bars: 1 cm. **C** Ex vivo BLI images of Ctrl (n = 12) and Lyve-1^−/−^ (n = 14) livers. Quantification of livers determined as region of interest at day14 after intrasplenic injection of B16F10 *luc2* melanoma cells (*P* = 0.0356, Mann–Whitney *U*-test). Color scale: min = 1.67 × 10^6^ p/sec/cm^2^ /sr; max = 2.46 × 10^7^ p/sec/cm^2^ /sr. **D** WT31 cells were injected into the tail vein and melanoma liver metastases (19.15 vs. 8.0, *P* = 0.0408, Mann–Whitney *U*-test) were counted in Ctrl (n = 13) and Lyve-1^−/−^ (n = 14) mice at day 19. Photographs of livers are displayed. Scale bars: 1 cm. **E** Ex vivo BLI images of Ctrl (n = 5) and Lyve-1^−/−^ (n = 4) livers. Quantification of livers determined as region of interest (*P* = 0.7508) 90 min after intrasplenic injection of B16F10 *luc2* melanoma cells. Color scale: min = 5.0 × 10^4^ p/sec/cm^2^ /sr; max = 6.6 × 10^4^ p/sec/cm^2^ /sr. Data information: * *P* < 0.05; ** *P* < 0.01; *** *P* < 0.001; **** *P* < 0.0001; *ns* not significant

The potential influence of Lyve-1 on the sinusoidal immunological microenvironment and the fact that LYVE-1 had been described to be able to mediate tumor cell adhesion in vitro [29], prompted us to study models of hepatic metastasis in Lyve-1-KO mice. For these experimental setups we chose relevant models of hepatic metastases of cancers that are known to be highly or weakly immunogenic, i.e. melanoma and colorectal carcinoma (CRC). Liver metastasis was studied by splenic injection of MC38 CRC cells in Lyve-1-KO and control mice. Here, no significant difference in the number of hepatic metastases could be observed (*P* = 0.5563) (Fig. 3A). On the contrary, when B16F10 *luc2* melanoma cells were inoculated in Lyve-1-KO and Ctrl mice by intrasplenic injection, liver colonization was significantly decreased (*P* = 0.0093) (Fig. 3B, C). Besides, this was confirmed in a second melanoma model as Lyve-1-KO also showed reduced hepatic colonization after spleen injection of WT31 melanoma as compared to Ctrl mice (*P* = 0.0164) (Additional file 1: Figure S3A). To further assess whether alteration of melanoma metastasis in Lyve-1-KO is liver-specific, our analysis was extended with a different route of melanoma cell administration. Therefore, WT31 melanoma, a cell line that in contrast to other murine melanoma cell lines induces not only lung but also hepatic metastasis after tail vein injection, was used [35]. While the number of hepatic metastases of WT31 melanoma was decreased in Lyve-1-KO compared to Ctrl mice (*P* = 0.0408) (Fig. 3D), the number of pulmonary WT31 melanoma metastases was not altered in Lyve-1-KO in comparison to Ctrl mice, indicating a specific role of Lyve-1 in liver but not lung colonization of melanoma (Additional file 1: Figure S3B). The reduction of hepatic metastatic foci of B16F10 *luc2* and WT31 melanoma in Lyve-1-KO as compared to Ctrl was confirmed by a significant reduction of the total macroscopic and microscopic tumor area in Lyve-1-KO with B16F10 *luc2* or WT31 metastases after spleen injection (Additional file 1: Figure S3C, D). When WT31 melanoma cells were injected i.v., sizes of hepatic metastases in general were smaller and only a trend towards smaller metastases in Lyve-1-KO was detected (Additional file 1: Figure S3C, D). As confirmation of the number of hepatic metastasis, no difference in the total metastatic area of MC38 CRC was detected between Lyve-1-KO and Ctrl (Additional file 1: Figure S3C, D). As Lyve-1 can be involved in tumor cell adhesion *in vitro*, initial tumor cell retention was also assessed in vivo by bioluminescence imaging in Lyve-1-KO and Ctrl mice 90 min after intrasplenic injection of B16F10 *luc2* melanoma cells. Here, similar intensities were observed in both groups indicating that Lyve-1 deficiency did not impair initial tumor cell retention of B16F10 *luc2* melanoma cells to the hepatic sinusoids (Fig. 3E).

### Analysis of morphology and vascularization of hepatic metastases

**Fig. 4:**
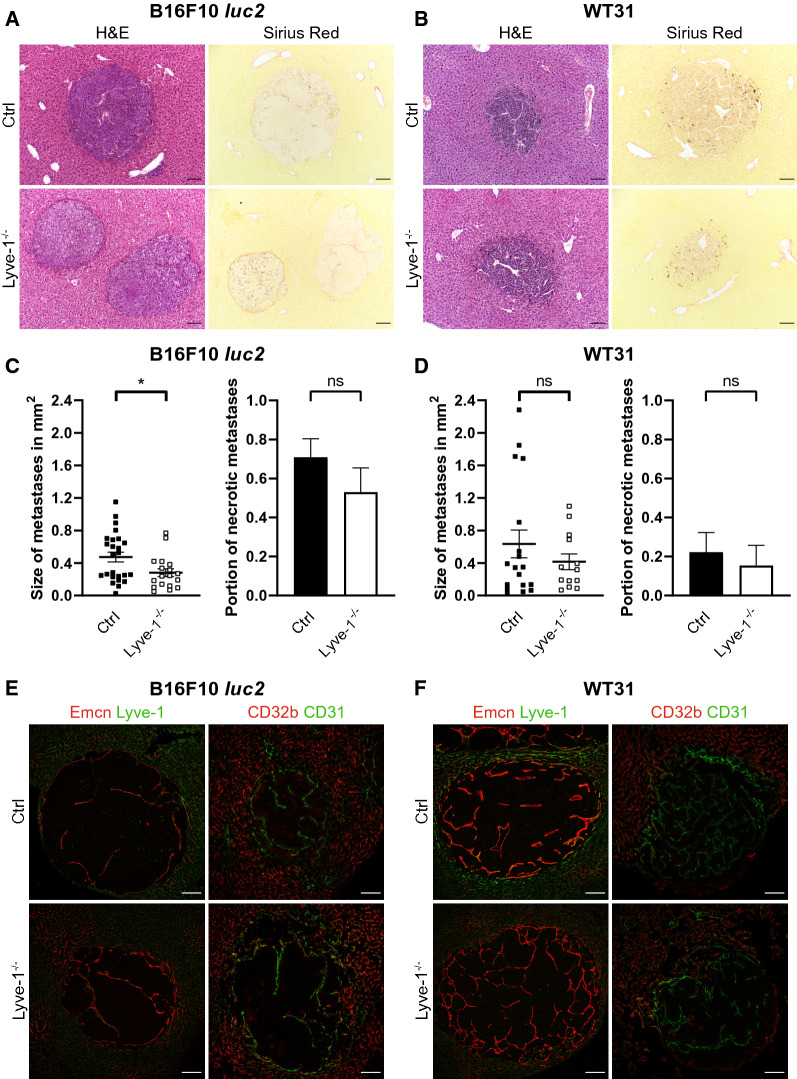
Microscopic analysis of B16F10 *luc2* or WT31 melanoma metastases in Lyve-1^−/−^ and Ctrl mice.** A** Images of H&E stainings of hepatic metastases of B16F10 *luc2* melanoma in Ctrl (n_met_ = 25, n = 7) and Lyve-1^−/−^ (n_met_ = 18, n = 5) livers. Images of Sirius red stainings of hepatic metastases of B16F10 *luc2* melanoma in Ctrl (n_met_ = 22, n = 8) and Lyve-1^−/−^ (n_met_ = 12, n = 4) livers. Scale bars: 100 µm. **B** Images of H&E stainings of hepatic metastases of WT31 melanoma in Ctrl (n_met_ = 18, n = 6) and Lyve-1^−/−^ (n_met_ = 13, n = 7) livers. Images of Sirius red stainings of hepatic metastases of WT31 melanoma in Ctrl (n_met_ = 14, n = 5) and Lyve-1^−/−^ (n_met_ = 10, n = 7) livers. Scale bars: 100 µm. **C** The size of liver metastases of B16F10 *luc2* melanoma in Ctrl (n_met_ = 25, n = 7) and Lyve-1^−/−^ (n_met_ = 18, n = 5) livers was measured (*P* = 0.0467, Mann-Whitney *U*-test) and area is presented in mm^2^. H&E stainings of hepatic B16F10 *luc2* melanoma metastases were evaluated for necrosis. The percentage of necrotic metastases referred to the total number of metastases was analyzed in Ctrl (n_met_ = 24, n = 6) and Lyve-1^−/−^ (n_met_ = 17, n = 5) (*P* = 0.3281, Mann-Whitney *U*-test). **D** The size of liver metastases of WT31 melanoma in Ctrl (n_met_ = 18, n = 6) and Lyve-1^−/−^ (n_met_ = 13, n = 7) livers was measured (*P* = 0.9528, Mann-Whitney *U*-test). The area is presented in mm^2^. H&E stainings of hepatic WT31 melanoma metastases were evaluated for necrosis. The percentage of necrotic metastases in relation to the total number of metastases in Ctrl (n_met_ = 18, n = 6) and Lyve-1^−/−^ (n_met_ = 13, n = 7) is presented (*P* = 0.6830, Mann-Whitney *U*-test). **E** Immunofluorescence stainings of hepatic B16F10 *luc2* melanoma metastases for Emcn and Lyve-1 in Ctrl (n_met_ = 20, n = 7) and Lyve-1^−/−^ (n_met_ = 17, n = 6) mice as well as for CD31 and CD32b in Ctrl (n_met_ = 12, n = 4) and Lyve-1^−/−^ (n_met_ = 6, n = 5) mice. Scale bars: 100 µm. **F** Immunofluorescence stainings of hepatic WT31 melanoma metastases for Emcn and Lyve-1 in Ctrl (n_met_ = 14, n = 5) and Lyve-1^−/−^ (n_met_ = 14, n = 7) mice as well as for CD31 and CD32b in Ctrl (n_met_ = 12, n = 7) and Lyve-1^−/−^ (n_met_ = 7, n = 5) mice. Scale bars: 100 µm. Data information: * *P* < 0.05; ** *P* < 0.01; *** *P* < 0.001; **** *P* < 0.0001; *ns* not significant

To study the underlying mechanisms, both B16F10 *luc2* and WT31 melanoma liver metastases were first assessed by histology. Hepatic metastases of B16F10 *luc2* and WT31 melanoma did not show obvious differences in their morphology, their growth patterns or associated fibrosis between Lyve-1-KO and Ctrl mice (Fig. 4A, B). While hepatic B16F10 *luc2* metastases were significantly larger in Ctrl livers as compared to Lyve-1-KO livers (*P* = 0.0467), the size of hepatic WT31 metastases was not significantly altered. Besides, the percentage of necrotic metastases of B16F10 *luc2* or WT31 hepatic metastases was unaltered when comparing Lyve-1-KO and Ctrl (Fig. 4C, D). As the loss of *Lyve-1* might impact on vascularization of the hepatic metastases, the vascularization of both B16F10 *luc2* and WT31 metastases was compared by immunofluorescent analysis of Emcn and Lyve-1 or of CD32b and CD31. Vascularization and intratumoral endothelial differentiation did not differ between melanoma metastases in Lyve-1-KO or Ctrl mice, except for the loss of Lyve-1 in Lyve-1-KO (Fig. 4E, F). To assess tumor cell proliferation and apoptosis, stainings for Ki67, cCasp-3 and DAPI were performed on hepatic metastases of Ctrl and Lyve-1-KO mice. The percentage of Ki67^+^ or Casp-3^+^ tumor cells did not significantly differ between hepatic melanoma metastases of Ctrl and Lyve-1-KO mice (Additional file 1: Figure S4A–D). Overall, these results indicated that tumor cell retention, tumor cell proliferation or apoptosis and alterations of vascularization were not responsible for the reduced susceptibility of the liver to melanoma metastasis in Lyve-1-KO.

### Characterization of the hepatic immune microenvironment

**Fig. 5:**
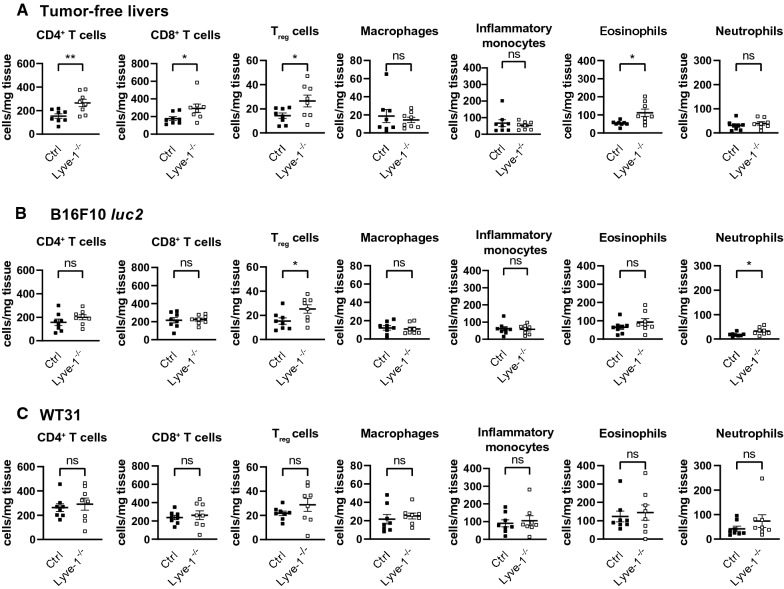
Analysis of the immune cell composition in livers of Ctrl and Lyve-1^−/−^ mice without metastases or with melanoma metastases of B16F10 *luc2* or WT31.** A** Flow cytometric analysis of liver immune cells including CD4^+^ T cells (*P* = 0.0094), CD8^+^ T cells (*P* = 0.0449), T_reg_ cells (*P* = 0.0389), macrophages (*P* = 0.7984, Mann–Whitney *U*-test), inflammatory monocytes (*P* = 0.7984, Mann–Whitney *U*-test), eosinophils (*P* = 0.0139) and neutrophils (*P* = 0.4159) was performed of whole untreated, tumor-free Ctrl and Lyve-1^−/−^ livers (female, n = 8/group). **B** Flow cytometric analysis of whole liver tissue with B16F10 *luc2* melanoma metastases of Ctrl and Lyve-1^−/−^ mice (female, n = 8/group) for CD4^+^ T cells (*P* = 0.2381), CD8^+^ T cells (*P* = 0.8248), T_reg_ cells (*P* = 0.0368), macrophages (*P* = 0.7209, Mann–Whitney *U*-test), inflammatory monocytes (*P* = 0.8420), eosinophils (*P* = 0.2228) and neutrophils (*P* = 0.0261). Analysis was performed on day 14. **C** Flow cytometric analysis of whole liver tissue with WT31 melanoma metastases of Ctrl and Lyve-1^−/−^ mice (female, n = 8/group) for CD4^+^ T cells (*P* = 0.6411), CD8^+^ T cells (*P* = 0.6405), T_reg_ cells (*P* = 0.2402), macrophages (*P* = 0.5676), inflammatory monocytes (*P* = 0.7209, Mann–Whitney *U*-test), eosinophils (*P* = 0.7984, Mann–Whitney *U*-test) and neutrophils (*P* = 0.2786, Mann–Whitney *U*-test). Analysis was performed on day 19. Data information: * *P* < 0.05; ** *P* < 0.01; *** *P* < 0.001; **** *P* < 0.0001; *ns* not significant

As melanoma is highly immunogenic, we reasoned that decreased melanoma metastasis formation in the liver may be mediated by increased anti-tumor immune activation. Besides, microarray gene expression analysis had also shown that immunological pathways were altered in premetastatic LSECs of Lyve-1-KO. Therefore, the immune cell composition of the hepatic niche was characterized in tumor-free, premetastatic livers and livers with established hepatic melanoma metastases of both Lyve-1-KO and Ctrl mice. To evaluate the extent to which Lyve-1 is expressed on hepatic macrophages, tumor-free and metastasized livers of Ctrl mice were stained for Lyve-1 and F4/80. In contrast to wound healing tissue, in Ctrl livers no definitive F4/80^+^/Lyve-1^+^ cells could be observed (Additional file 1: Figure S5A–D). This is in line with previously published data showing that F4/80^+^ Lyve-1^+^ cells were seen in connective tissues of visceral organs and tumors, but not in hepatic sinusoids [25, 26]. As a role of Lyve-1 during leukocyte adhesion to lymphatic EC is described [28], the subtypes of infiltrating immune cells in tumor-free and metastasized Ctrl and Lyve-1-KO livers were analyzed in detail by flow cytometry (Fig. 5, Additional file 1: Figure S6A). Tumor-free Lyve-1-KO livers showed increased numbers of CD4^+^ T cells (*P* = 0.0094), CD8^+^ T cells (*P* = 0.0449), regulatory T cells (T_reg_) (*P* = 0.0389) and eosinophils (*P* = 0.0139) compared to Ctrl livers, whereas other immune cell subtypes remained unchanged (Fig. 5A; Additional file 1: Figure S6B). In livers with established B16F10 *luc2* melanoma metastases a significant difference in the numbers of T_reg_ cells (*P* = 0.0368) and neutrophils (*P* = 0.0261) was found in livers of Lyve-1-KO in contrast to Ctrl (Fig. 5B, Additional file 1: Figure S6C). Livers with WT31 melanoma metastases did not differ in hepatic immune cell composition between Lyve-1-KO and Ctrl (Fig. 5C, Additional file 1: Figure S6D). Besides, immunofluorescence stainings of CD45^+^ Ly6C^+^ cells, CD45^+^ Ly6C^+^ Ly6G^+^ cells and CD4^+^ FoxP3^+^ cells showed no altered distribution in the peritumoral liver tissue of Lyve-1-KO and Ctrl with hepatic metastases of B16F10 *luc2* or WT31 (Additional file 1: Figure S7A–F).

Altogether, constitutive loss of Lyve-1 did not influence hepatic morphology, sinusoidal endothelial differentiation or angiocrine functions of LSECs. However, endothelial scavenging functions were impaired, as hyaluronan plasma levels and levels of Factor XIII and CA2 were altered in Lyve-1-KO. Moreover, iron deposition was observed in macrophage subpopulations. Liver metastasis as a pathological process was influenced in a tumor-specific fashion as hepatic metastasis of cutaneous melanoma, but not CRC, was reduced upon knockout of Lyve-1. As tumor cell retention, proliferation and vascularization were not altered, our findings indicate that this is likely due to increased hepatic leukocyte subpopulations that mediate initial tumor cell rejection by an enhanced premetastatic tumor immune surveillance.

## Discussion

LYVE-1/Lyve-1 is expressed on lymphatic endothelium, subsets of vascular endothelial cells, for example LSECs, and on subgroups of macrophages, such as certain tumor-infiltrating macrophages. Its molecular functions are also diverse, as it has been implied in endocytosis and scavenging, as well as in adhesion and migration of immune and tumor cells.

LSECs are the major endothelial scavenger cell population and responsible for clearance of circulating blood factors. LYVE-1/Lyve-1 was initially assumed to act as a scavenging receptor of hyaluronan (HA) [21, 27]. However, previous studies in Stab1 and Stab2 deficient mice have clearly identified Stab2 as the major scavenger receptor responsible for the clearance of HA [20]. In contrast to investigations by Gale et al. with an alternative knockout approach of *Lyve-1* [32], we found mildly, but consistently elevated plasma levels of hyaluronan in animals with *Lyve-1* deficiency. Notably, hyaluronan plasma levels were not analyzed in the original description of the *Lyve-1* knockout mice designed by Huang et al. and used in this study [31]. The difference between the two knockout strains first described by Gale et al. and Huang et al. may result from discrepancies of the genetic background, age or environmental factors, such as food or stress levels. Nonetheless, in comparison to Stab2 KO mice HA levels were about 3 to fourfold lower in Lyve-1-KO plasma indicating that Stab2 is the major scavenger receptor responsible for clearance of HA in the blood, while Lyve-1 appears to have a context-dependent smaller role that is mostly compensated by Stab2. One can hypothesize that functions of Lyve-1 might also be compensated by its homologue CD44. Unfortunately, only tissue levels of hyaluronic acid after carrageenan-induced paw edema have been analyzed in Lyve-1/CD44 double knockout mice and did not show any differences compared to Lyve-1-KO and wild-type mice [33]. Interestingly, Lyve-1/CD44 double knockout mice do not present any overt phenotype. Comprehensive analysis of clearance functions of Lyve-1 by plasma proteomics revealed only CA2 as potential selective protein ligand accumulating in the blood of Lyve-1-KO mice. Factor XIII on the other hand was less abundant in blood plasma of Lyve-1-KO. This indicates that Lyve-1 appears to be involved in the control of blood plasma composition to a smaller extent in comparison to Stab1 and Stab2.

During tumor metastasis, hepatic endothelial cells represent the first barrier interacting with arriving cancer cells via surface receptors like E-selectin, ICAM1 or Clec4g [14–16]. Besides, adhesion of hyaluronan-expressing tumor cells to endothelial cells is mediated by Lyve-1 [29]. Contrastingly, high levels of plasma HA strongly decreased metastasis of B16F10 melanoma to the lungs by reduction of tumor cell adhesion to the pulmonary capillaries [38]. Of note, Hirose et al. identified that high levels of HA did not impact melanoma cell invasion, migration or proliferation. However, in our liver colonization model no differences in initial melanoma cell retention were detected among Lyve-1-KO and Ctrl mice. This might be explained by tumor cell-type and context-dependency, as well as by cellular and molecular differences of *in vivo* and in vitro models of tumor cell adhesion. Transdifferentiation and capillarization of LSECs can also influence hepatic metastasis e.g. via alterations of tumor cell adhesion [13]. However, as Lyve-1-KO showed no signs of capillarization or alterations of angiocrine metabolic functions, this cannot explain reduced melanoma metastasis in Lyve-1-KO livers.

Alterations in hepatic metastasis of melanoma but not CRC indicate that the anti-tumor immune response may be altered in Lyve-1-KO. Lyve-1 is involved in hyaluronan-dependent leukocyte trafficking in lymphatic EC [28, 39] and is expressed on subtypes of macrophages. However, in our Lyve-1-KO mouse model, there were almost no macrophages in the liver detected that co-express Lyve-1. Neither by flow cytometry significant differences in the number of hepatic macrophages were revealed. This is in line with the study of Zheng et al. that did not find an expression of Lyve-1 in macrophages of visceral organs [25].

Interestingly, loss of Lyve-1 led to iron deposition in F4/80^+^ macrophage subpopulations and decreased levels of iron in the plasma. In general, iron metabolism plays a decisive role in macrophages and inflammatory processes [40]. Macrophages exert a special function during iron metabolism and are of special importance for recycling of iron from senescent erythrocytes and the export into the plasma [41]. Hereby, the iron exporter ferroportin1 is critically involved as a macrophage-specific loss of ferroportin1 leads to hepatic iron depositions and increases inflammatory responses [42]. These iron depositions appear like the ones in Lyve-1-KO mice leading us to hypothesize that Lyve-1 may also be involved during hepatic iron turnover. Apart from that, hepatic iron homeostasis is critically controlled by angiocrine secretion of Bmp2 and Bmp6 by LSECs [6, 7]. However, deficiency of either Bmp2 or Bmp6 leads to a different pattern of iron deposition and a microarray analysis of isolated LSECs revealed no significant differences in the levels of Bmp2 or Bmp6 between Lyve-1-KO and Ctrl mice. Besides, there is accumulating evidence that iron deposition in macrophages promotes a switch towards a pro-inflammatory phenotype during inflammatory conditions, such as non-alcoholic steatohepatitis, anti-cancer immunity or wound healing [43–45]. Regarding liver metastasis, application of ferumoxytolof, an iron supplement, effectively prevented liver colonization of small-cell lung cancer [46].

Altogether, the significant reduction in the number of B16F10 *luc2* and WT31 melanoma liver metastases might be due to an increased premetastatic pro-inflammatory microenvironment in the liver. This is substantiated by microarray analyses of isolated LSECs showing regulation of relevant inflammatory pathways. Although there were no significant changes in the number of CD4^+^ and CD8^+^ T cells in livers of Lyve-1 KO mice bearing established melanoma metastases, tumor-free livers of Lyve-1 KO mice showed significant increased numbers of CD4^+^ and CD8^+^ T cells, T_reg_ cells and eosinophils. These alterations may result from either modulation of leukocyte influx via LSEC or modulation of leukocyte efflux via lymphatic EC. The protective role of T cells for liver metastasis, especially CD8^+^ T cells, is highlighted by various studies. Hepatic immunomodulation by α-melittin-nanoparticles increases numbers of CD4^+^ and CD8^+^ T cells, which protect from hepatic metastasis [47]. Besides, clinical studies of CRC long-term survivors with liver metastases and a clinical case of metastatic uveal melanoma reveal a prognostic benefit of higher numbers of CD8^+^ T cells in the liver [48, 49]. Our results identify an enhanced premetastatic hepatic immune microenvironment which might promote initial tumor cell rejection and consequently decreased hepatic melanoma colonization, as initial tumor cell retention in mice with *Lyve-1* deficiency was unaltered. The liver specific effect of Lyve-1 on melanoma metastasis could be proven by the intravenous injection route of WT31 melanoma cells, as deficiency of *Lyve-1* led to a significant protection against hepatic but not pulmonary melanoma colonization. Last, the tumor-specific differences in hepatic metastasis in our model might be explained by varying immunogenicity of the CRC cell line MC38 as compared to the two melanoma models.

To treat liver metastasis of CM immune checkpoint inhibition (ICI) or targeted therapies (BRAF/ MEK inhibitors) are state-of-the art options. However, liver metastasis of CM goes along with a poor prognosis for both therapy response to ICI [47] and targeted therapy [50]. Therefore, the microenvironment of the hepatic niche needs further attention and new therapeutic targets must be evaluated. Due to the decreased hepatic metastasis in two melanoma models and the increased premetastatic pro-inflammatory state in Lyve-1-KO, Lyve-1 might be an interesting therapeutic target [30] to further boost therapy response to ICI.

## Conclusions

Liver metastasis of melanoma is a poor prognostic factor. Lyve-1 deficiency protected from hepatic melanoma metastasis in an orthotopic injection model. While hepatic endothelial differentiation and angiocrine functions were unaltered, loss of Lyve-1 generated a premetastatic pro-inflammatory microenvironment characterized by increased CD4^+^, CD8^+^ T cells and T_reg_ cells and by iron deposition in hepatic macrophages. Therefore, Lyve-1 should be further investigated as promising target of the hepatic microenvironment to improve response to current therapies.

## Supplementary Information

**Additional file 1: **Supplementary Figures. **Figure S1**. Additional phenotypic characterization of Ctrl and Lyve-1^-/-^ mice. Analysis of the iron homeostasis. Further analyses of hyaluronan levels by different ELISAs and by age. **A**. Body length (female, *P* = 0.5438, n = 5-6/group; male, *P* = 0.7832, n = 5/group) measured of Ctrl and Lyve-1^-/-^ mice.** B**. Levels of ALT (female, *P* = 0.3176, n = 8-9/group), AST (female, *P* = 0.1270, Mann-Whitney *U*-test, n = 6-7/group), GLDH (female, *P* = 0.2095, n = 7-9/group) in blood plasma of Ctrl and Lyve-1^-/-^ mice are displayed.** C**. Iron concentration in liver (female, *P* = 0.4110, n = 3/group), plasma (female, *P* = 0.0387, n = 6/group), and Hepcidin concentration in plasma (female, *P* = 0.3747, n = 6-8/group) of Ctrl and Lyve-1^-/-^ mice. **D**. Hyaluronan concentration in plasma of Ctrl and Lyve-1^-/-^ mice in the age of ***a*** 6 weeks (female, *P* < 0.0001, n = 6/group), ***b*** 8 weeks (female, *P* < 0.0001, n = 6/group) and c 10 weeks (female, *P* < 0.0001, n = 6/group) measured with Hyaluronan DuoSet ELISA (DY3614-05, R&D Systems, Minneapolis, MN, USA). **E**. Hyaluronan concentration in plasma of Ctrl and Lyve-1^-/-^ mice in the age of ***a*** 12 weeks (female, *P* = 0.0022, Mann-Whitney *U*-test, n = 6/group) and ***b*** 14 weeks (female, *P* = 0.0079, Mann-Whitney *U*-test, n = 5/group) measured with Hyaluronan Enzyme-Linked Immunosorbent Assay (K-1200, Echelon Biosciences, Salt Lake City, UT, USA). Data information: * *P* < 0.05; ** *P* < 0.01; *** *P* < 0.001; **** *P* < 0.0001; ns = not significant. **Figure S2**. Analysis of platelet counts, functions and hemostasis in Ctrl or Lyve-1^-/-^ mice. **A**. Platelet count (female, *P* = 0.7831, n = 5-9/group), thromboelastography (TEG) R [min] (male, *P* = 0.0536, n = 5/group), TEG K [min] (male, *P* >0.9999, Mann-Whitney *U*-test, n = 5/group), TEG Angle [degree] (male, *P* = 0.3413, Mann-Whitney *U*-test, n = 5/group) and TEG MA [mm] (male, *P* = 0.1211, n = 5/group) quantified in blood of Ctrl and Lyve-1^-/-^ mice. **B**. Activated partial thromboplastin time (aPTT) (female, *P* = 0.1040, n = 6/group), INR (female, *P* = 0.5647, n = 4-5/group) and thrombin time (female, *P* = 0.8084, n = 6/group) measured in blood of Ctrl and Lyve-1^-/-^ mice. Data information: * *P* < 0.05; ** *P* < 0.01; *** *P* < 0.001; **** *P* < 0.0001; ns = not significant. **Figure S3**. Quantification of hepatic metastases after intrasplenic of WT31 cells as well as quantification of lung metastases after intravenous injection of WT31 cells. Measurement of microscopic and macroscopic metastatic area. **A**. After spleen injection of WT31 melanoma cells the numbers of macroscopic visible hepatic metastases (57.6 vs. 7.6, P = 0.0164) were quantified in Ctrl (n = 10) and Lyve-1^-/-^ (n = 5) mice at day 21. The ROUT method was used to detect outliers. Two outliers were excluded in the Lyve-1^-/-^ group. Representative photographs of livers with melanoma metastases are shown. Scale bars: 1 cm. **B**. WT31 cells were injected into the tail vein and melanoma lung metastases (41.92 vs. 59.71, *P* = 0.1343) were counted in Ctrl (n = 13) and Lyve-1^-/-^ (n = 14) mice at day 19. Photographs of lungs are displayed. Scale bars: 1 cm. **C**. The hepatic macroscopic metastatic area in relation to the total liver area was analyzed per mouse. MC38 hepatic metastatic area (*P* = 0.6336) in Ctrl (n = 5) and Lyve-1^-/-^ (n = 5). B16F10 *luc2* hepatic metastatic area (*P* = 0.0175, Mann-Whitney *U*-test) in Ctrl (n = 12) and Lyve-1^-/-^ (n = 14). Hepatic metastatic area after intrasplenic injection of WT31 melanoma (*P* = 0.0256) in Ctrl (n = 10) and Lyve-1^-/-^ (n = 5). Hepatic metastatic area after i.v. injection of WT31 melanoma (*P* = 0.4502, Mann-Whitney *U*-test) in Ctrl (n = 13) and Lyve-1^-/-^ (n = 14). **D**. The hepatic microscopic metastatic area in relation to the total liver area was analyzed per mouse. MC38 hepatic metastatic area (*P* = 0.9675) in Ctrl (n = 5) and Lyve-1^-/-^ (n = 5). B16F10 *luc2* hepatic metastatic area (*P* = 0.0068, Mann-Whitney *U*-test) in Ctrl (n = 6) and Lyve-1^-/-^ (n = 9). Hepatic metastatic area after intrasplenic injection of WT31 melanoma (*P* = 0.0186, Mann-Whitney *U*-test) in Ctrl (n = 8) and Lyve-1^-/-^ (n = 5). Hepatic metastatic area after i.v. injection of WT31 melanoma (*P* = 0.8443, Mann-Whitney *U*-test) in Ctrl (n = 12) and Lyve-1^-/-^ (n = 14). Data information: * *P* < 0.05; ** *P* < 0.01; *** *P* < 0.001; **** *P* < 0.0001; ns = not significant. **Figure S4**. Analysis of cell proliferation or apoptosis in hepatic melanoma metastases of Ctrl and Lyve-1^-/-^ mice. **A**. Immunofluorescence stainings of hepatic B16F10 *luc2* melanoma metastases for Ki67 and Emcn in Ctrl (n_met_ = 14, n = 4) and Lyve-1^-/-^ (n_met_ = 12, n = 5) mice as well as for cleaved Caspase-3 (cCasp-3) and Emcn in Ctrl (n_met_ = 14, n = 4) and Lyve-1^-/-^ (n_met_ = 12, n = 5) mice. Scale bars: 100 µm. **B**. Immunofluorescence stainings of hepatic WT31 melanoma metastases for Ki67 and Emcn in Ctrl (n_met_ = 16, n = 7) and Lyve-1^-/-^ (n_met_ = 11, n = 6) mice as well as for cCasp-3 and Emcn in Ctrl (n_met_ = 9, n = 5) and Lyve-1^-/-^ (n_met_ = 6, n = 5) mice. Scale bars: 100 µm. **C**. Percentage of Ki67^+^DAPI^+^ cells (*P* = 0.2972, Mann-Whitney *U*-test) in Ctrl (n_met_ = 14, n = 4) and Lyve-1^-/-^ (n_met_ = 12, n = 5) mice or cCasp-3^+^DAPI^+^ cells (*P* = 0.7808, Mann-Whitney *U*-test) in Ctrl (n_met_ = 14, n= 4) and Lyve-1^-/-^ (n_met_ = 12, n = 5) mice in relation to total DAPI^+^ melanoma cells counted in hepatic B16F10 *luc2* melanoma metastases. **D**. Percentage of Ki67^+^DAPI^+^ cells (*P* = 0.9594) in Ctrl (n_met_ = 16, n = 7) and Lyve-1^-/-^ (n_met_ = 11, n = 6) mice or cCasp-3^+^DAPI^+^ cells (P = 0.8637, Mann-Whitney *U*-test) in Ctrl (n_met_ = 9, n = 5) and Lyve-1^-/-^ (n_met_ = 6, n = 5) mice in relation to total DAPI^+^ melanoma cells counted in hepatic WT31 melanoma metastases. Data information: * *P* < 0.05; ** *P* < 0.01; *** *P* < 0.001; **** *P* < 0.0001; ns = not significant. **Figure S5**. Analysis of F4/80^+^Lyve-1^+^ cells in tumor-free and metastasized Ctrl livers. **A**. Immunofluorescence stainings for F4/80 and Lyve-1 in 3-day-old skin wound healing tissue of wild type mice (n = 2). Wounds were previously induced by 4mm punch biopsy under anaesthesia with Isoflurane. **B**. Immunofluorescence stainings of F4/80 and Lyve-1 in untreated, tumor-free Ctrl and Lyve-1 ^-/-^ liver tissue (female, n = 6/group). Scale bars: 100 µm. **C**. Immunofluorescence stainings of F4/80 and Lyve-1 in livers with metastases of B16F10 *luc2* melanoma cells in Ctrl (n_met_ = 10, n = 6) and Lyve-1^-/-^ mice (n_met_ = 6, n = 5). **D**. Immunofluorescence stainings of F4/80 and Lyve-1 in livers with metastases of WT31 melanoma cells of Ctrl (n_met_ = 12, n = 5) and Lyve-1^-/-^ mice (n_met_ = 12, n = 5). **Figure S6**. Gating strategy applied to identify indicated hepatic immune cell populations in both tumor-free and metastasized livers by flow cytometry. Additional analyses of dendritic cells, NK cells, NKT cells and gamma delta (gd) T cells in tumor-free and metastasized Lyve-1^-/-^ livers by flow cytometry. **A**. Single-cell suspensions of liver tissue were analyzed by flow cytometry. Cells gated as single/live CD45^+^ were further defined as: B cells (CD3ε^neg^CD19^+^), γδT T cells (CD19^neg^CD3ε^+^TCRγδ^+^), NKT cells (CD3ε^+^TCRβ^+^NK1.1^+^), CD8^+^ T cells (CD3ε^+^TCRβ^+^NK1.1^neg^CD8a^+^), CD4^+^ T cells (CD3ε^+^TCRβ^+^NK1.1^neg^CD4^+^), T_reg_ (CD3ε^+^TCRβ^+^NK1.1^neg^CD4^+^FoxP3^+^), NK cells (CD3ε^neg^CD19^neg^NKp46^+^NK1.1^+^Eomes^+^); myeloid cells are first gated as Lymphoid^neg^ CD3ε^neg^CD19^neg^NKp46^neg^NK1.1^neg^), and then further defined as neutrophils (CD11b^+^Ly6G^+^), eosinophils (CD11b^+^Ly6G^int^SiglecF^+^), DCs (CD11b^+^Ly6G^neg^SiglecF^neg^CD11c^high^MHC-II^high^), macrophages (non-DCs F4/80^+^Ly6C^neg^), and inflammatory monocytes (non-DCs F4/80^+^Ly6C^+^). Representative end-gates with simplified ascending gating paths for each population are shown. **B**. Flow cytometric analysis of liver immune cells including dendritic cells (*P* = 0.2459), NK cells (*P* = 0.1165), NKT cells (*P* = 0.0973) and gdT cells (*P* = 0.0653) was performed of whole untreated, tumor-free Ctrl and Lyve-1^-/-^ livers (female, n = 5-8/group). **C**. Flow cytometric analysis of whole liver tissue with B16F10 *luc2* melanoma metastases of Ctrl and Lyve-1^-/-^ mice (female, n = 5-8/group) for dendritic cells (*P* = 0.2624), NK cells (*P* = 0.6368), NKT cells (*P* = 0.1412) and gdT cells (*P* = 0.1668). **D**. Flow cytometric analysis of whole liver tissue with WT31 melanoma metastases of Ctrl and Lyve-1^-/-^ mice (female, n = 8/group) for dendritic cells (*P* = 0.3006), NK cells (*P* = 0.7971), NKT cells (*P* = 0.3127) and gdT cells (*P* = 0.8224). Data information: * *P* < 0.05; ** *P* < 0.01; *** *P* < 0.001; **** *P* < 0.0001; ns = not significant. **Figure S7**. Unaltered numbers of myeloid-derived suppressor cells and T_regs_ in peritumoral liver tissue of Ctrl and Lyve-1^-/-^ with B16F10 *luc2* or WT31 metastases. **A**. Immunofluorescence stainings of peritumoral hepatic tissue for DAPI, CD45 and Ly6C as well as for DAPI, CD45 and Ly6C/Ly6G in Ctrl (n = 4) and Lyve-1^-/-^ (n = 5) mice with B16F10 *luc2* metastases. Scale bars: 100 µm. **B**. Immunofluorescence stainings of peritumoral hepatic tissue for DAPI, CD45 and Ly6C as well as for DAPI, CD45 and Ly6C/Ly6G in Ctrl (n = 6) and Lyve-1^-/-^ (n = 5) mice with WT31 metastases. Scale bars: 100 µm. **C**. Quantification of peritumoral CD45^+^Ly6C^+^ cells (*P* = 0.099) or CD45^+^Ly6C^+^Ly6G^+^ cells (*P* = 0.3896) in livers of Ctrl (n= 4) and Lyve-1^-/-^ (n = 5) mice with metastases of B16F10 *luc2*. **D**. Quantification of peritumoral CD45^+^Ly6C^+^ cells (*P* = 0.7117) or CD45^+^Ly6C^+^Ly6G^+^ cells (*P* = 0.1967) in livers of Ctrl (n= 6) and Lyve-1^-/-^ (n = 5) mice with metastases of WT31. **E**. Quantification of peritumoral CD4^+^FoxP3^+^ cells (*P* = 0.1852) in livers of Ctrl (n= 8) and Lyve-1^-/-^ (n = 7) mice with B16F10 *luc2* metastases. **F**. Quantification of peritumoral CD4^+^FoxP3^+^ cells (P = 0.9984) in livers of Ctrl (n = 9) and Lyve-1^-/-^ (n = 8) mice with WT31 metastases. Data information: * *P* < 0.05; ** *P* < 0.01; *** *P* < 0.001; **** *P* < 0.0001; ns = not significant.

**Additional file 2: **Supplementary Methods.

**Additional file 3: **Supplementary Tables. **Table S1**. Primer sequences for genotyping.

## Data Availability

The microarray data are accessible in the Gene Expression Omnibus database (GEO) by GSE199055. The proteomics data are available via ProteomeXchange with identifier PXD032717.

## Abbreviations

AEC: 3-Amino-9-ethylcarbazole
ALT: Alanine aminotransferase
aPTT: Activated partial thromboplastin time
Arg1: Arginase 1
AST: Aspartate aminotransferase
BLI: Bioluminescence imaging
Bmp: Bone morphogenetic protein 2
CA2: Carbonic anhydrase 2
cCasp-3: Cleaved Caspase-3
CM: Cutaneous melanoma
Ctrl: Control/ wild-type
Cy3: Cyanine 3
CYP: Cytochrome P450
DC: Dendritic cell
EC: Endothelial cell
Emcn: Endomucin
Gd T-cells: Gamma delta T-cells
GLDH: Glutamate dehydrogenase
Glul: Glutamine synthetase
H&E: Hematoxylin and eosin
HRP: Horseradish peroxidase
Icam-1: Intracellular adhesion molecule 1
IF: Immunofluorescence
IHC: Immunohistochemistry
Inflam. Mo: Inflammatory monocytes
KO: Knockout
Lama4: Laminin, alpha 4
LSECs: Liver sinusoidal endothelial cells
LYVE-1: Human lymphatic vessel endothelial hyaluronan receptor-1
Lyve-1: Mouse lymphatic vessel endothelial hyaluronan receptor-1
Lyve-1^−^^/^^−^ / Lyve-1-KO: B6.129S1-*Lyve1*^*tm1Lhua*^/J/global Lyve-1 knockout
n: Number of mice analyzed
n_met_: Number of metastases analyzed
NK cell: Natural killer cell
NK T cell: Natural killer T cell
ns: Not significant
OCT: Cryo embedding matrix
PBS: Phosphate-buffered saline
PFA: Paraformaldehyde
PT: Prothrombin time
RhBg: Rhesus blood group-associated B glycoprotein
sec: Seconds
SEM: Standard error of mean
Stab1: Stabilin-1
Stab2: Stabilin-2
TAMs: Tumor-associated macrophages
TEG: Thromboelastography
T_reg_ cell: Regulatory T cell
