# Supplementary material for: Lyve-1 deficiency enhances the hepatic immune microenvironment entailing altered susceptibility to melanoma liver metastasis
Source: Cancer Cell Int. 2022 Dec 10;22:398. doi: 10.1186/s12935-022-02800-x (PMC9741792; doi:10.1186/s12935-022-02800-x)

Suppl. Figure 1

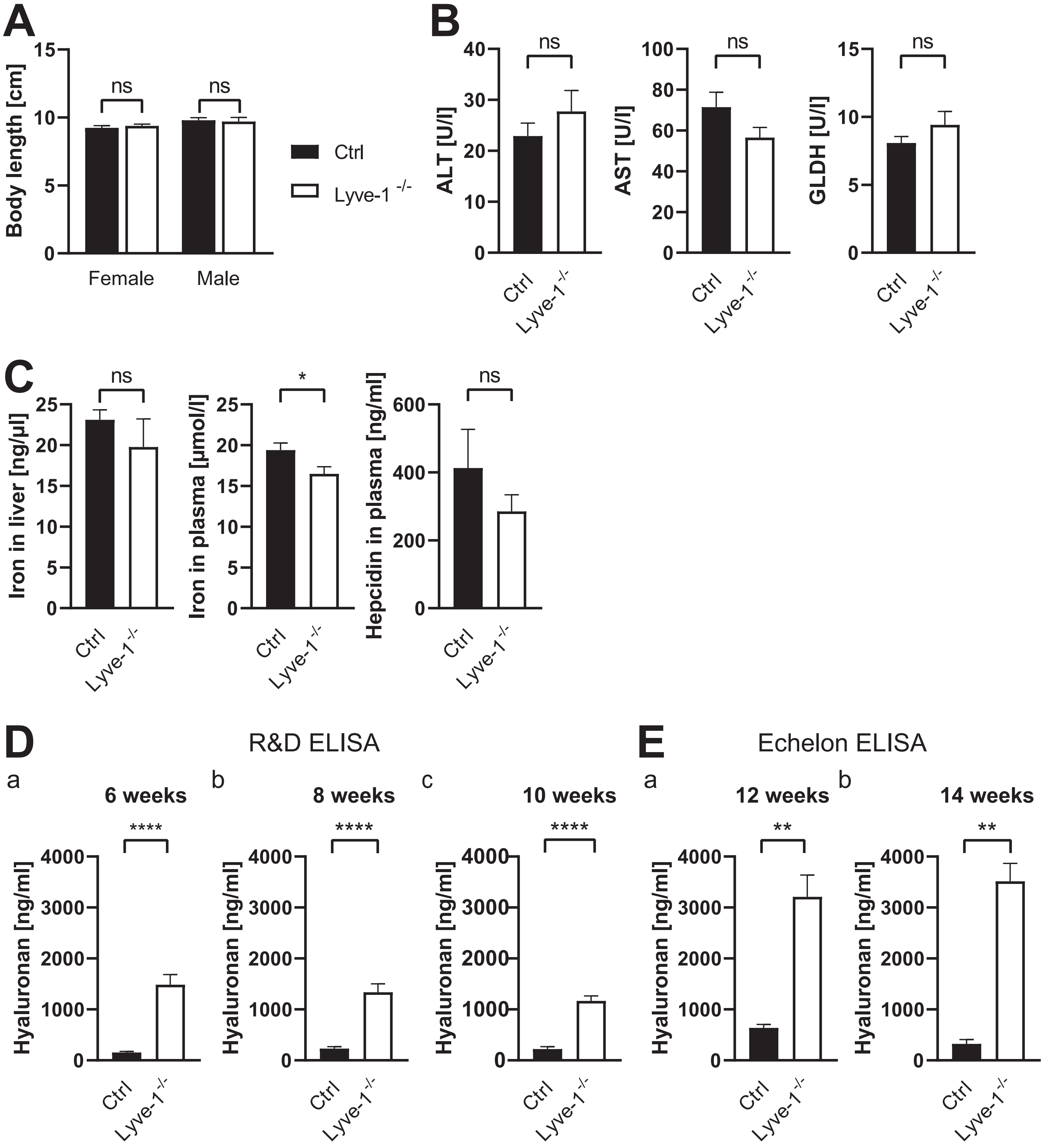

Suppl. Figure 2

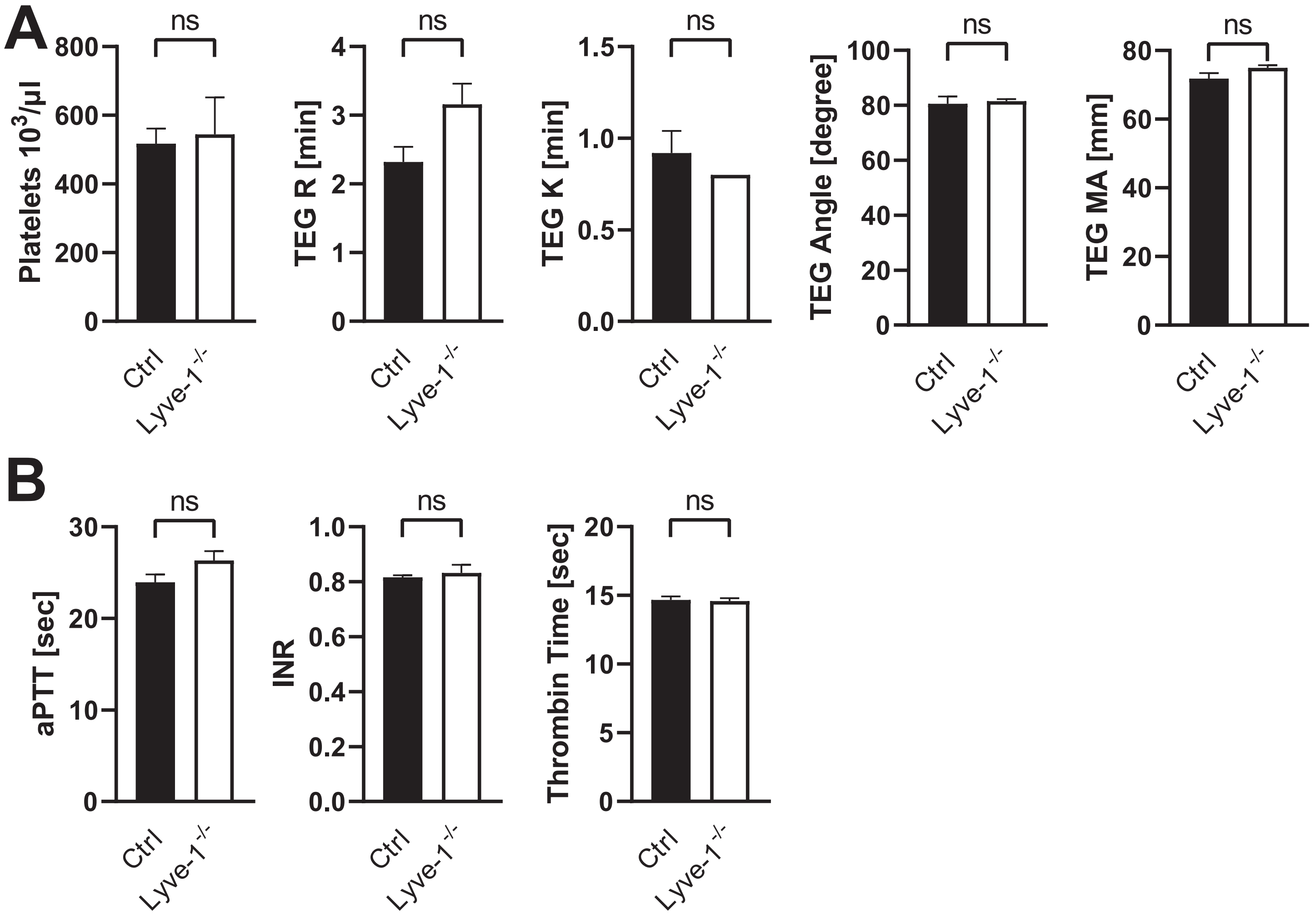

Suppl. Figure 3

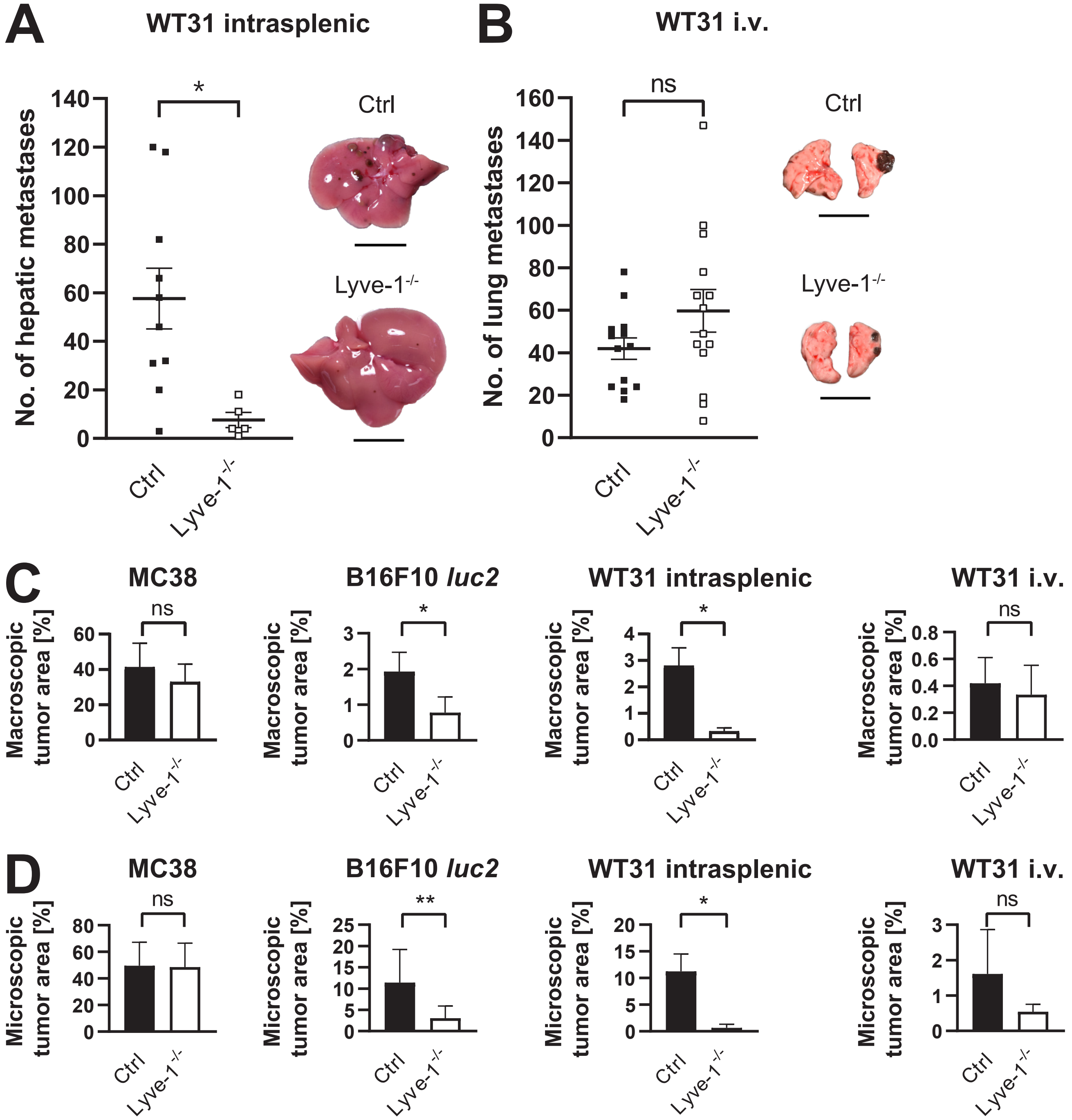

Suppl. Figure 4

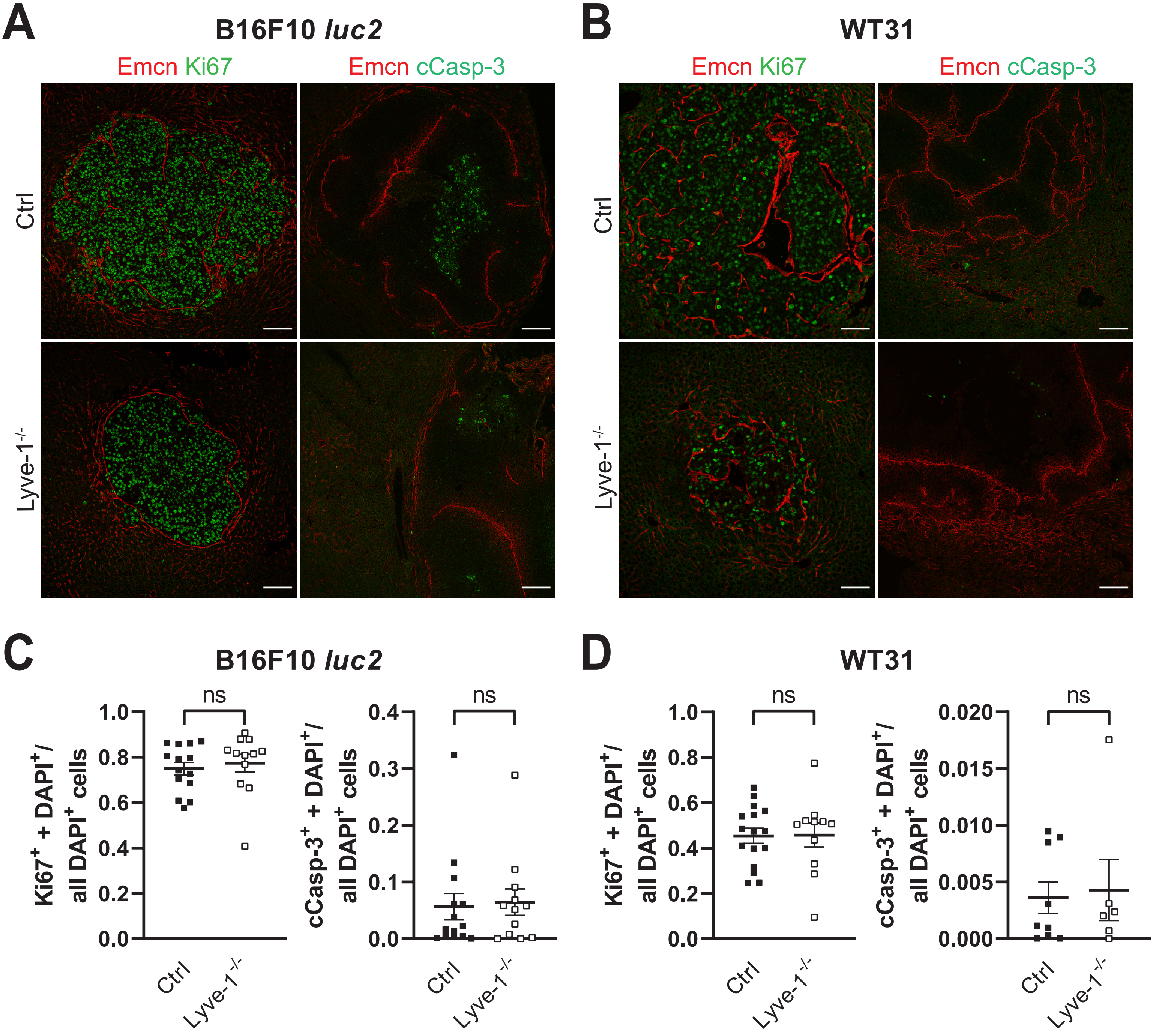

Suppl. Figure 5

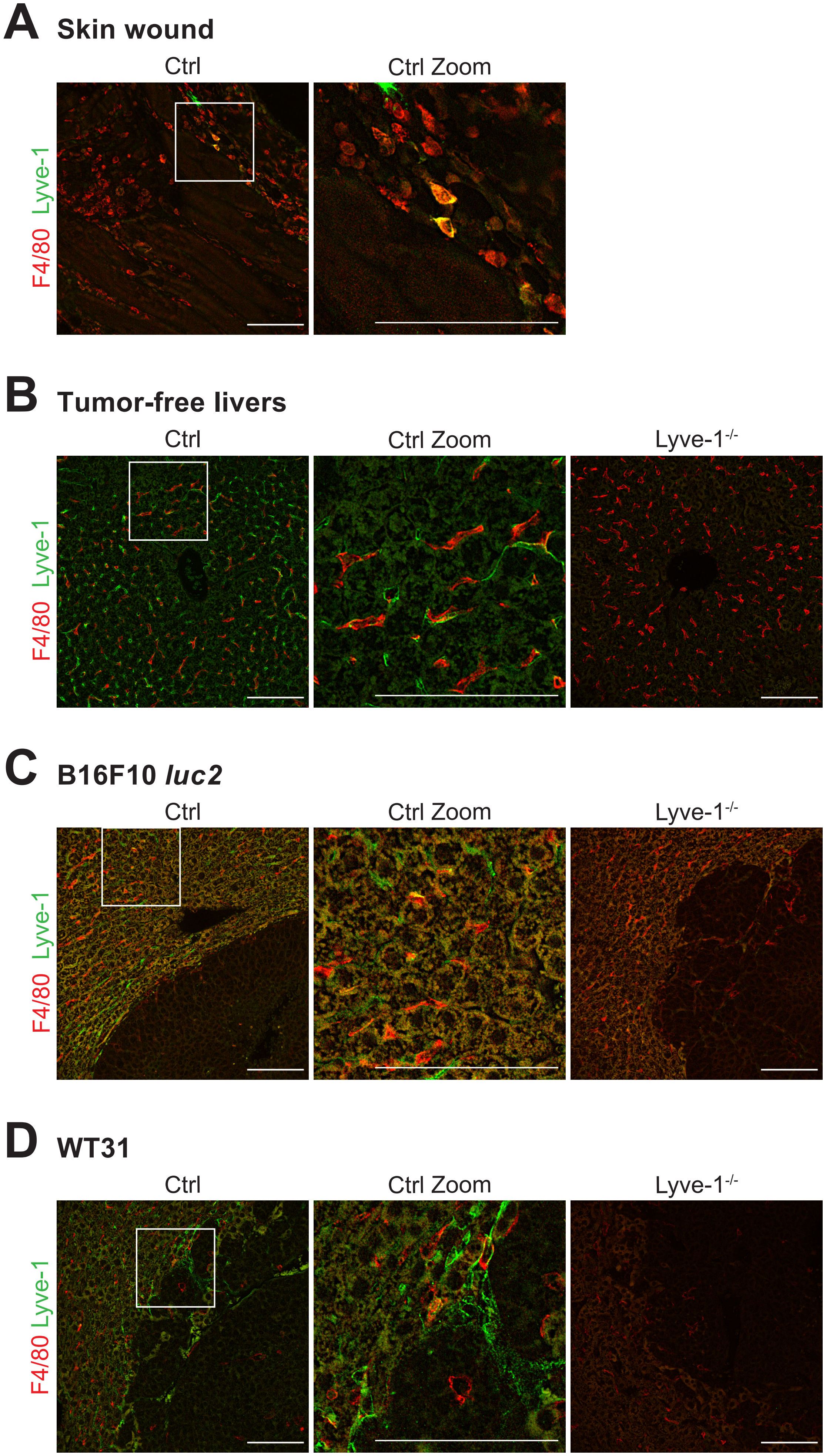

Suppl. Figure 6

A

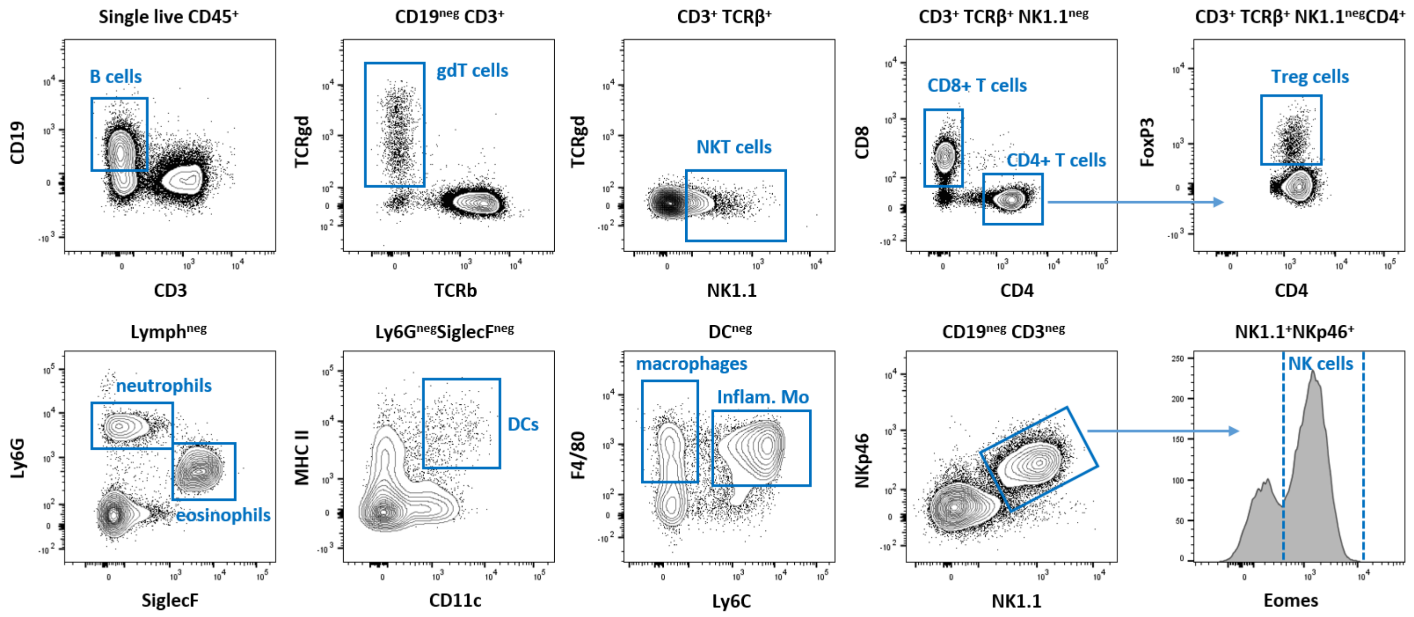

B Tumor-free livers

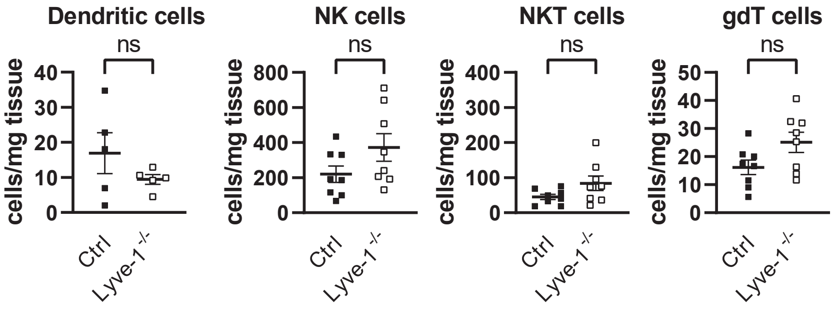

C B16F10 *luc2*

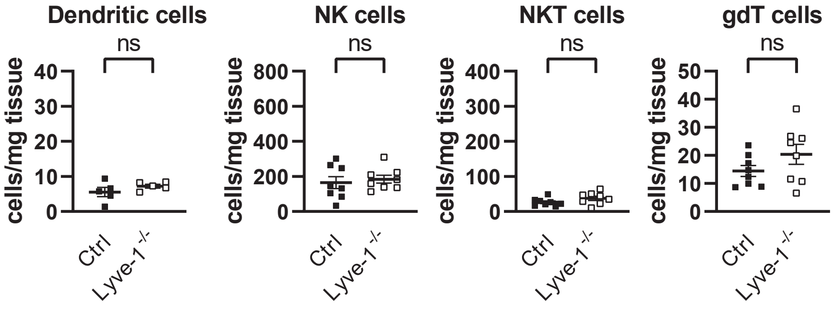

D WT31

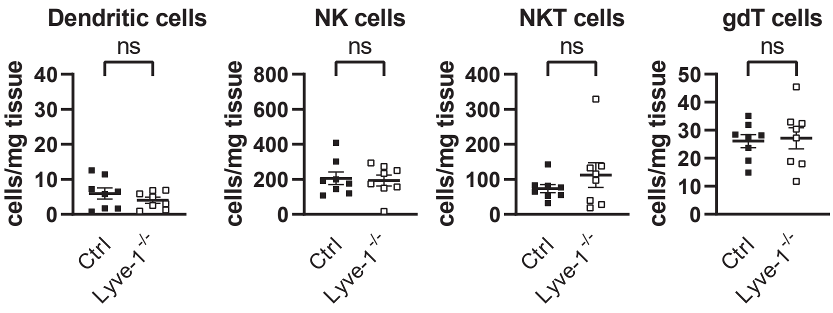

Suppl. Figure 7

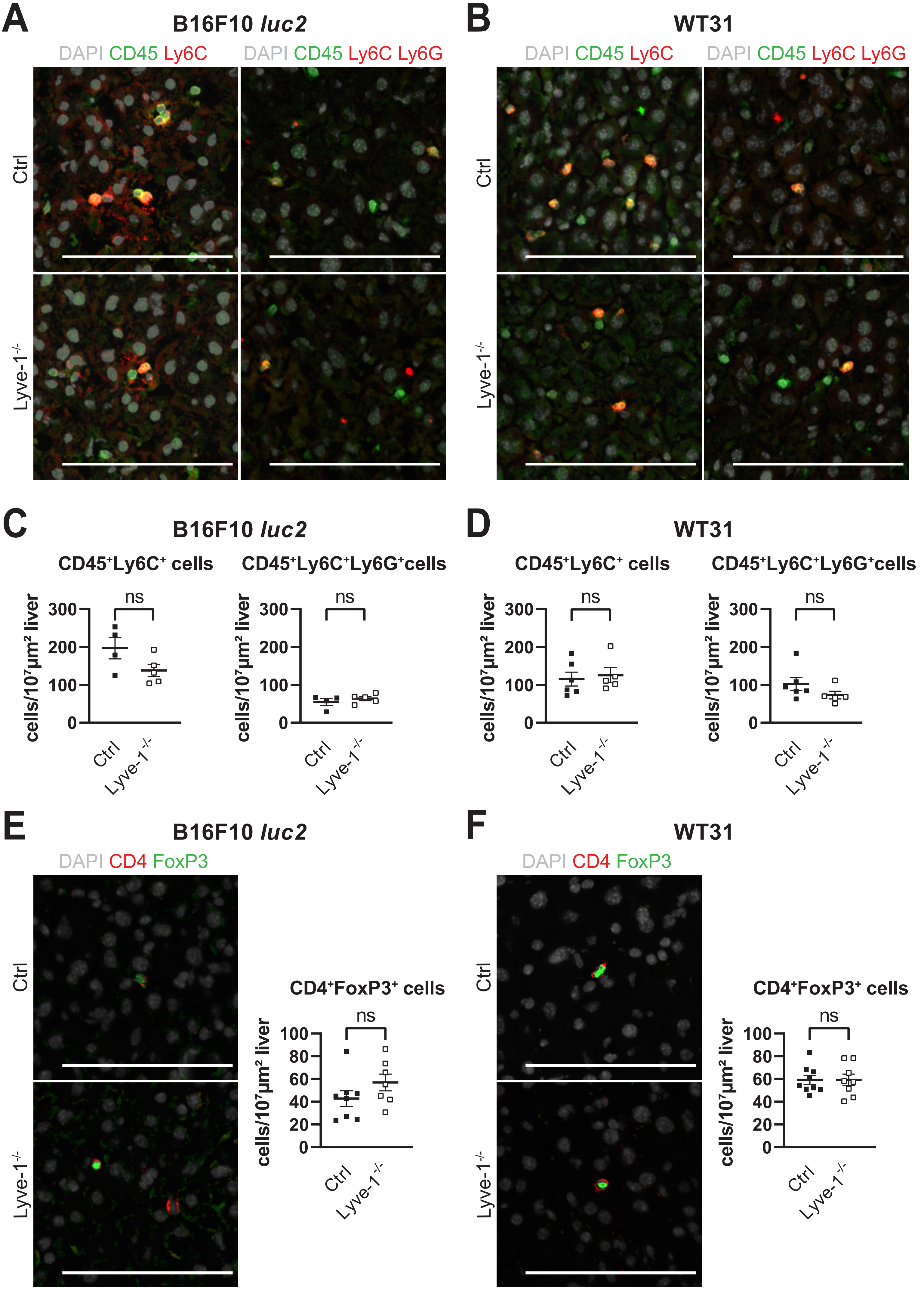

Supplement: Supplementary file 1 — Additional file 1: Supplementary Figures. Figure S1. Additional phenotypic characterization of Ctrl and Lyve-1-/- mice. Analysis of the iron homeostasis. Further analyses of hyaluronan levels by different ELISAs and by age. A. Body length (female, P = 0.5438, n = 5-6/group; male, P = 0.7832, n = 5/group) measured of Ctrl and Lyve-1-/- mice. B. Levels of ALT (female, P = 0.3176, n = 8-9/group), AST (female, P = 0.1270, Mann-Whitney U-test, n = 6-7/group), GLDH (female, P = 0.2095, n = 7-9/group) in blood plasma of Ctrl and Lyve-1-/- mice are displayed. C. Iron concentration in liver (female, P = 0.4110, n = 3/group), plasma (female, P = 0.0387, n = 6/group), and Hepcidin concentration in plasma (female, P = 0.3747, n = 6-8/group) of Ctrl and Lyve-1-/- mice. D. Hyaluronan concentration in plasma of Ctrl and Lyve-1-/- mice in the age of a 6 weeks (female, P < 0.0001, n = 6/group), b 8 weeks (female, P < 0.0001, n = 6/group) and c 10 weeks (female, P < 0.0001, n = 6/group) measured with Hyaluronan DuoSet ELISA (DY3614-05, R&D Systems, Minneapolis, MN, USA). E. Hyaluronan concentration in plasma of Ctrl and Lyve-1-/- mice in the age of a 12 weeks (female, P = 0.0022, Mann-Whitney U-test, n = 6/group) and b 14 weeks (female, P = 0.0079, Mann-Whitney U-test, n = 5/group) measured with Hyaluronan Enzyme-Linked Immunosorbent Assay (K-1200, Echelon Biosciences, Salt Lake City, UT, USA). Data information: * P < 0.05; ** P < 0.01; *** P < 0.001; **** P < 0.0001; ns = not significant. Figure S2. Analysis of platelet counts, functions and hemostasis in Ctrl or Lyve-1-/- mice. A. Platelet count (female, P = 0.7831, n = 5-9/group), thromboelastography (TEG) R [min] (male, P = 0.0536, n = 5/group), TEG K [min] (male, P >0.9999, Mann-Whitney U-test, n = 5/group), TEG Angle [degree] (male, P = 0.3413, Mann-Whitney U-test, n = 5/group) and TEG MA [mm] (male, P = 0.1211, n = 5/group) quantified in blood of Ctrl and Lyve-1-/- mice. B. Activated partial thromboplastin time (aPTT) (female, [file 12935_2022_2800_MOESM1_ESM.pdf]
