# Supplementary material for: Lyve-1 deficiency enhances the hepatic immune microenvironment entailing altered susceptibility to melanoma liver metastasis
Source: Cancer Cell Int. 2022 Dec 10;22:398. doi: 10.1186/s12935-022-02800-x (PMC9741792; doi:10.1186/s12935-022-02800-x)
Supplement: Supplementary file 2 — Additional file 2: Supplementary Methods. [file 12935_2022_2800_MOESM2_ESM.docx]

**ADDITIONAL METHODS**

**Analysis of blood parameters**Blood samples were taken after anesthesia with Isofluran from the retrobulbar venous plexus as final blood withdrawal in lithium heparin tubes (Microvette 500 Lithium-Heparin, 20.1345, Sarstedt, Nümbrecht, Germany). For plasma samples, blood was separated (centrifugation at 7000 x g for 7 minutes). EDTA tubes (Microvette APT 200 K3EDTA, 20.1288, Sarstedt, Nümbrecht, Germany) were utilized for platelet count and citrate tubes (Micro Tubes with attached PE push cap closure, 41.1506.002, Sarstedt, Nümbrecht, Germany) for coagulation parameters and platelet function.

**Measurement of hepatic parameters, hepcidin and hyaluronan in plasma**

Alanine transaminase (ALT), aspartate transaminase (AST) and glutamate dehydrogenase (GLDH) in plasma was determined by a Cobas C 311 (Roche Diagnostics, Rotkreuz, Switzerland) according to the manufacturer’s protocol. For measuring hepcidin levels in plasma the Hepcidin Murine-Compete ELISA Kit (HMC-001, Intrinsic LifeSciences, La Jolla, CA, USA) was used according to the manufacturer’s recommendations using an Infinite m200 pro plate reader (Tecan Group, Männedorf, Switzerland). For the measurement of hyaluronan concentration in plasma, the Hyaluronan DuoSet ELISA (DY3614-05, R&D Systems, Minneapolis, MN, USA) and Hyaluronan Enzyme-Linked Immunosorbent Assay (K-1200, Echelon Biosciences, Salt Lake City, UT, USA) were used according to the manufacturers’ instructions with the help of Infinite m200 pro plate reader (Tecan Group, Männedorf, Switzerland).

**Measurement of platelet count, iron, coagulation parameters and platelet function in blood and plasma**

All analyses were performed in accredited laboratories under DIN ISO EN 15189 conditions. Platelet count was determined using EDTA whole blood on the Hematology Analyzer XN-10 (Sysmex K.K., Kōbe, Japan). Iron determinations were conducted using a fully automated colorimetric ferrozine-based assay on the Siemens Vista Dimension 1500 (Siemens, Eschborn, Germany) platform from mouse plasma. The manufacturer states a limit of quantification of 0.4 μmol/L and an analytical measuring range of 0.4 - 179.0 µmol/L. Plasmatic coagulation measurements were performed from citrate plasma on a CS 5100 (Sysmex K.K., Kōbe, Japan) device. To be able to work with small sample volumes, the supernatants were transferred to Hitachi Cups and the micro method of the instrument was used. Activated partial thromboplastin time was determined with the coagulometric assay using Dade® Actin® FS activator. For the determination of PT and thrombin time, Dade® Innovin® and Thromboclotin® (all: Siemens Healthineers AG, Erlangen, Germany) were used receptively. Thromboelastography (TEG) as a surrogate parameter for platelet function was conducted using TEG 5000 analyzer (Haemonetics, Boston, MA, USA). Whole citrate blood samples were activated with CaCl2 and Kaolin (Haemonetics, 07-004, Boston, MA, USA). The assay was performed for at least 30 minutes. All assays were applied as stated by the respective manufacturers without further modification or validation.

**Plasma proteomics**

**Plasma proteomics protein digestion**

Plasma samples were lysed in 4% SDS and reduced (TCEP, 10 mM) and alkylated (CAA, 20 mM) for 60 min at 45°C and digested using the SP3 digestion technique ^1^ using a one to one mixture of two different magnet beads (Sera-Mag(TM) Magnetic Carboxylate Modified Particles (Hydrophobic) #GE44152105050250, Sera-Mag(TM) Magnetic Carboxylate Modified Particles (Hydrophylic) #GE24152105050250). 3 µL of washed bead mixture (3 x PBS wash) was added to 20 µg of protein lysate and acetonitrile was added to a final concentration of 50%. The lysate was incubated for 8 min on a magnet and the liquid was removed. Proteins bound to the beads, were washed twice with 160 µL acetonitrile using the CyBio Flex liquid handler system (Analytic Jena, Germany). Beads were dried for 5 minutes and 20 µL of a digestion solution containing 0.1 µg Trypsin (Sigma-Aldrich, USA) and 0.1 µg Lys-C (Wako) in 20 mM HEPES pH=8.5 was added for overnight incubation at 37°C. The solution was incubated for 5 min in an ultrasonic batch to dissolve beads. Peptides were cleaned-up by 2 wash steps using 200 µL acetonitrile. Beads were incubated in 2% freshly prepared DMSO (Sigma-Aldrich, USA) and dried to complete dryness in a speed vac concentrator (Eppendorf, Germany) and further processed using the StageTip technique ^2^. Prior to LC-MS/MS measurements, peptides were re-suspended in 10 µL 2% acetonitrile and 2% formic acid.

**Liquid chromatography and mass spectrometry**

LC–MS/MS instrumentation consisted out of an Easy nLC-1200 (Thermo Fisher Scientific, USA) coupled via a nanospray-ionization source to a Exploris 480 (Thermo Fisher Scientific, USA) mass spectrometer. A binary buffer system consisting out of solvent A and B (buffer A: 0.1% formic acid and buffer B: 0.1% formic acid in 80% acetonitrile) was utilized for peptide separation. The in-house packed column length was 40 cm (ID = 75 µm). The column was filled with PoroShell C18 2.7-µm (Agilent Technologies, USA) beads and a column oven controlled the temperate at 50 °C (PRSO-V2, Sonation). The buffer B percentage was linearly raised from 5% to 27% within 69 min and further increased to 65% within 10 min. Buffer B content was then increased to 95% within 6 min. The column was washed at 95% B for 10 min. The total method time was 95 min per samples but the acquisition stopped after 90 min followed by 5 min column washing at 95% B. All samples were measured in random order. The mass spectrometer operated in data independent acquisition mode. MS1 spectra were acquired at a resolution of 120,000 and an AGC target of 1 × 106. In total, 48 DIA windows were acquired at an isolation m/z range of 15 Th and the isolation windows overlapped by 2 Th, covering a mass range from 320 to 1040 m/z. Resolution of MS2 spectra was set to 15,000 at 200 m/z using a maximal injection time of 22 ms and stepped normalized collision energies (NCE) of 26, 28, 30. The samples were measured using the FAIMS interface at compensation value of -50 using the following temperature settings to decrease the FAIMS resolution: Inner electrode temperature: 99.5°C. and the outer electrode temperature was set to 85°C. The FAIMS gas was set to 0 L/min (off).

**Proteomics data analysis and data processing**

Raw files were acquired using DIA (Data independent acquisition) mode. We utilized DIA-NN version 1.8 ^3^. First a library was generated using plasma proteomic samples acquired raw files using the Mus Musculus Uniport (downloaded december 2020) reference proteome fasta file (available within the PRIDE repository). The options ‘Fasta digest for library-free search / library generation’ and ‘Deep learning-based spectra, RTs and IMs prediction’ were enabled. A single miss cleavage was allowed and N-term M excision and carbamidomethylation at cysteine residues were enabled as fixed modification. ‘Mass accuracy’, ‘MS1 accuracy’, and ‘Scan window’ options were set to 0 (automatic interference). The neuronal network classifier was to ‘Double-pass mode’. The Precursor m/z range was defined to 300 – 1200. Further settings were ‘Protein interence’ = Isoform IDs and the quantification strategy was set to ‘Robust LC (high accuracy)’. The generated library containing 5216 potentially precursors was then used to re-analyze the project specific raw files. Quantities matrices were exported and the protein group specific output was used in the downstream analysis. To identify significantly different proteins, a two-sided t-test was applied using log2 transformed MaxLFQ intensities calculated within the DIA-NN software. The FDR was controlled to 5% using a permutation-based approach in the Perseus software ^4^. Results were visualized using the Instant Clue software ^5^.

The mass spectrometry proteomics data have been deposited to the ProteomeXchange Consortium via the PRIDE ^6^ partner repository with the dataset identifier PXD032717.

**Tissue dissection, cryopreservation and paraffin embedding**

After anesthetizing the mice in Isofluran CP (21311, WDT, Garbsen, Germany), mice were sacrificed by cervical dislocation. For validation of the genotype a part of the tail was cut. Organs were excised, put either first into OCT (Sakura Finetek Europe B.V. KvK, Alphen aan den Rijn, Netherlands) or were directly shock frozen in liquid nitrogen. Furthermore, tissue was placed in 4% formaldehyde solution (P087, Carl Roth, Karlsruhe, Germany) for at least 24 hours. Tissue in formaldehyde solution was then embedded in paraffin according to standard protocols.

For flow cytometry, livers were first perfused with Dulbecco’s Phosphate Buffered Saline (14190094, Thermo Fisher Scientific, Waltham, MA, USA) via the portal vein. Then the livers were put into tubes (227261, Greiner Bio One, Kremsmünster, Austria) filled with DMEM Medium (61965059, Gibco™, Thermo Fisher Scientific, Waltham, MA, USA) and bedded on ice.

**Histology, immunofluorescence and immunohistochemistry**

Paraffin embedded tissue was cut into 3 µm thick sections. Then routine stainings H&E, Prussian blue and Sirius red were performed according to standard protocols of the manufacturer.
Immunofluorescence stainings were either performed on paraffin embedded tissue or on cryoconserved tissue. After cutting of paraffin embedded tissue into 3 µm thick slides, the tissue was deparaffinized and rehydrated according to standard protocols. Antigen retrieval was performed by using HIER citrate buffer pH 6.0 (ZUC028-500, Zytomed Sytems, Berlin, Germany) or HIER EDTA Buffer pH 8.0 (ZUC040-500, Zytomed Sytems, Berlin, Germany) or HIER T-EDTA Buffer pH 9.0 (ZUC029-500, Zytomed Sytems, Berlin, Germany) for either 30 or 45 minutes at 95°C. Cryoconserved tissue was cut into 8 µm thick slices and was air dried. For fixation 4% paraformaldehyde (PFA) (0335, Carl Roth, Germany) was put on the tissue for ten minutes. Afterwards PBS with 5% normal donkey serum (017-000-121, Dianova, Hamburg, Germany) was placed on the tissue for blocking for 30 minutes.
The primary antibody, either diluted in purchased Dako antibody diluent (S202230-2, Agilent Technologies, Santa Clara, USA) or diluted in self-produced antibody diluent (PBS substituted with normal donkey serum), was put on the tissue at 4 °C over night. Following three washing steps, the secondary antibody was applied for one hour at room temperature. Dako fluorescent mounting medium (S302380-2, Dako, Agilent technologies, Santa Clara, CA, USA) was used to mount the sections.
Immunohistological stainings was performed on paraffin embedded tissue after deparaffinization, rehydration and antigen retrieval by first blocking the tissue with Dako peroxidase solution (S2023, Dako, Agilent Technologies, Santa Clara, USA). The primary antibody diluted in Dako antibody diluent was incubated at 4°C over night. After three washing steps, the secondary antibody conjugated with horseradish peroxidase (HRP) was applied for one hour at room temperature. After adding Dako AEC substrate chromogen (K3464, Dako, Agilent Technologies, Santa Clara, CA, USA) for 10 minutes, an adapted Prussian blue staining was conducted in 1% [hydrochloric](https://dict.leo.org/englisch-deutsch/hydrochloric) [acid](https://dict.leo.org/englisch-deutsch/acid) (Dr. K. Hollborn & Söhne, EG-Nr. 231-595-7, Ch.-B.0419, Leipzig, Germany) mixed with 2% kalium ferrocyanide solution (Dr. K. Hollborn & Söhne, EG-Nr. 237-722-2, Ch.-B.0419, Leipzig, Germany). Then the slides were put into Mayer’s hemalum solution (1.09249.2500, Merck, Darmstadt, Germany) for counterstaining. Dako aqueous mounting medium (S3025, Dako, Agilent Technologies, Santa Clara, CA, USA) was used for mounting the slices.

**Antibodies**

**Primary antibodies:** Rabbit anti-Lyve-1 (103-PA50S, ReliaTech, Wolfenbüttel, Germany), goat anti-Lyve-1 (AF2125, R&D Systems, Minneapolis, MN, USA), rat anti-Endomucin (14-5851-82, Thermo Fisher Scientific, Waltham, MA, USA), rat anti-F4/80 (123102, BioLegend, San Diego, CA, USA), rat anti-CD31 (DIA-310, Dianova, Hamburg, Germany), goat anti-CD32/CD16 (AF1460, R&D Systems, Minneapolis, MN, USA), rabbit anti-Stabilin-2 peptide 15 antibody^36^, rabbit anti-Glutamine Synthetase (G2781, Sigma-Aldrich, St. Louis, MO, USA), rabbit anti-Arginase 1 (3668S, Cell Signaling Technology, Danvers, MA, USA), rabbit anti-Icam-1 (10020-1-AP, Proteintech, Rosemont, IL, USA), goat anti-Lama4 (AF3837, R&D Systems, Minneapolis, MN, USA), rabbit anti-Desmin (ab32362, Abcam, Cambridge, UK), goat anti-RhBg (PA5-19369, Thermo Fisher Scientific, Waltham, MA, USA), rabbit anti-β-catenin (ab32572, Abcam, Cambridge, UK), rabbit anti-CYP2E1 (AB1252, Sigma-Aldrich, St. Louis, MO, USA), rabbit anti-Cleaved Caspase-3 (9661, Cell Signaling Technology, Danvers, MA, USA), rabbit anti-Ki67 (ab16667, Abcam, Cambridge, UK), rabbit anti-GAPDH (2118S, Cell Signaling Technology, Danvers, MA, USA), rat anti-CD4 (100402, BioLegend, San Diego, CA, USA), rabbit anti-FoxP3 (ab215206, Abcam, Cambridge, UK), rabbit anti-CD45 (ab10558, Abcam, Cambridge, UK), rat anti-Ly6C (ab15627, Abcam, Cambridge, UK) and rat anti-Ly6C and anti-Ly6G (ab25377, Abcam, Cambridge, UK).

**Secondary antibodies:** Cy3-AffiniPure Donkey Anti-Rat IgG (712-165-153, Dianova, Hamburg, Germany), Cy3-AffiniPure Donkey Anti-Rabbit IgG (711-165-152, Dianova, Hamburg, Germany), Cy3-AffiniPure Donkey Anti-Goat IgG (705-165- 147, Dianova, Hamburg, Germany), Alexa Fluor 647-AffiniPure Donkey Anti-Rat IgG (712-605-153, Dianova, Hamburg, Germany), Alexa Fluor 488- AffiniPure Donkey Anti-Rabbit IgG (711-545- 152, Dianova, Hamburg, Germany) were used for immunofluorescence. HRP-conjugated Goat Anti-Rat IgG (AP136P, Sigma-Aldrich, St. Louis, MO, USA) was used for immunohistochemistry. For immunoblot the Donkey anti-Rabbit IgG, HRP Conjugated (NA934, Merck, Darmstadt, Germany) and Donkey Anti-Goat IgG Antibody, HRP conjugate (A16005, ThermoFisher Scientific, Waltham, MA, USA) were utilized.

**Image acquisition and processing**

To capture images of routine, immunohistochemistry and immunofluorescence stainings an Eclipse Ni-E motorized upright microscope with CFI Plan Apochromat Lambda series objective lenses (10x, 20x, 40x, 60x) was utilized. The microscope is equipped with an Intensilight Epifluorescence Illuminator. A DS-Ri2 high-definition color camera was used to acquire images of routine and immunohistochemistry stainings. To capture immunofluorescence stainings a DS-Qi2 high-definition monochrome camera was applied. NIS-Elements AR 5.02 software (Nikon Instruments, Tokyo, Japan) was used. The images were processed by NIS-Elements AR 5.02 by applying background reduction, deconvolution and focusing. Fiji ImageJ 2.0.0-rc-68/1.53d software was used for image optimization.

To quantify the expression of Lyve-1 and Emcn we applied Fiji ImageJ 2.0.0-rc-68/1.53d software and assessed the area of immunofluorescence signal in the image with reference to the whole area of the image. Five independently chosen pictures were analyzed per mouse and the mean value of the area in percent was calculated. For co-localization analysis of proliferation marker such as Ki67 and apoptosis marker such as cleaved Caspase-3 (cCasp-3) with DAPI the NIS-Elements 5.02 software was used.

Prussian blue stainings were as well acquired with a PANNORAMIC 250 Slide Scanner (3DHISTECH Ltd., Budapest, Hungary) using a 20x objective. Pannoramic Viewer software (3DHISTECH Ltd., Budapest, Hungary) was used. For quantification of iron dots on Prussian blue stainings, five representative areas were chosen in the 20x magnification and the iron dots were counted by sight. A plausibility check was performed optically for each quantification.

Besides, immunofluorescence stainings of CD45+Ly6C, CD45+Ly6C+Ly6G, CD4+Foxp3 as well as H&E stainings were scanned with an automated slide scanner Axio Scan.Z1 (Zeiss, Jena, Germany). Whole liver sections were analyzed by Imaris 9.9 (Oxford Instruments, Oxon UK). Briefly, the whole liver tissue was analyzed as DAPI positive area by calculating a surface. Then the metastatic area was marked manually. Double-positive immune cells were detected by a threshold-based spot calculation. Thresholds were set for two immune cell markers and the DAPI signal. The number of positive spots was counted in the peritumoral liver tissue and normalized to the peritumoral liver area.

Moreover, Fiji ImageJ 2.0.0-rc-68/1.53d software was applied to analyze the microscopic tumor area in H&E stainings. Both the metastatic areas and the total liver area of one section were marked manually. The metastatic areas were summed up as total metastatic area and set in relation to the total liver area.

Macroscopic tumor area was quantified by measuring the total metastatic and hepatic area in photographs of livers (front and back surface) with Fiji ImageJ and calculating the percentage of the respective areas.

**Immunoblot**

RIPA lysis and extraction buffer (R0278, Sigma-Aldrich, St. Louis, MO, USA), and protease inhibitor cocktail (cOmplete, 11836170001, Roche, Merck, Darmstadt, Germany) was used for homogenization of liver samples in a Precellys Evolution tissue homogenizer (Bertin Technologies, Montigny-le-Bretonneux, France). For determination of the total protein, the homogenized liver was centrifuged and the supernatant was measured with DC Protein Assay (5000112, Bio-Rad Laboratories, Hercules, CA, USA). Before running electrophoresis 2x Laemmli buffer (1610737, Bio-Rad Laboratories) substituted with 5% β-mercaptoethanol (M3148-100ML, Sigma-Aldrich, St. Louis, MO, USA) was added to the samples and the mix was boiled. For electrophoresis a 4–20 % polyacrylamide gel (Mini-PROTEAN® TGX Stain-Free™ Protein Gels, 4568093, BioRad, Hercules, CA, USA) was used, here 100 µg of protein was inserted into the wells. With the help of a Trans-Blot Turbo Transfer System (1704150, Bio-Rad Laboratories, Hercules, CA, USA) the transfer of protein samples to a PVDF membrane (1620177, BioRad, Hercules, CA, USA) was performed in a semi-dry electrophoretic transfer. Blocking was conducted with 5% skim milk/PBS for 30 minutes. Then the membranes were incubated with the Lyve-1 primary antibody for 24 hours at 4°C. After three washing steps with PBS with 0,1 % Tween (P7949, Sigma-Aldrich, St. Louis, MO, USA), the corresponding secondary HRP-conjugated-antibody was incubated for one hour at room temperature. After incubating the membrane in Immobilon Forte Western HRP substrate (WBLUF0500, Merck, Millipore, Darmstadt, Germany), Azure Imager c400 (Azure Biosystems, Dublin, CA, USA) was used for detecting chemiluminescence signals of respective proteins and standard. After that the whole process was repeated with the GAPDH primary and respective secondary HRP-conjugated antibody.

**References:**

1. Hughes CS, Moggridge S, Müller T, et al. Single-pot, solid-phase-enhanced sample preparation for proteomics experiments. *Nat Protoc*. 01 2019;14(1):68-85. doi:10.1038/s41596-018-0082-x

2. Rappsilber J, Mann M, Ishihama Y. Protocol for micro-purification, enrichment, pre-fractionation and storage of peptides for proteomics using StageTips. *Nat Protoc*. 2007;2(8):1896-906. doi:10.1038/nprot.2007.261

3. Demichev V, Messner CB, Vernardis SI, Lilley KS, Ralser M. DIA-NN: neural networks and interference correction enable deep proteome coverage in high throughput. *Nat Methods*. 01 2020;17(1):41-44. doi:10.1038/s41592-019-0638-x

4. Tyanova S, Temu T, Sinitcyn P, et al. The Perseus computational platform for comprehensive analysis of (prote)omics data. *Nat Methods*. 09 2016;13(9):731-40. doi:10.1038/nmeth.3901

5. Nolte H, MacVicar TD, Tellkamp F, Krüger M. Instant Clue: A Software Suite for Interactive Data Visualization and Analysis. *Sci Rep*. 08 23 2018;8(1):12648. doi:10.1038/s41598-018-31154-6

6. Perez-Riverol Y, Csordas A, Bai J, et al. The PRIDE database and related tools and resources in 2019: improving support for quantification data. *Nucleic acids research*. Jan 8 2019;47(D1):D442-d450. doi:10.1093/nar/gky1106
