# Supplementary material for: Lyve-1 deficiency enhances the hepatic immune microenvironment entailing altered susceptibility to melanoma liver metastasis
Source: Cancer Cell Int. 2022 Dec 10;22:398. doi: 10.1186/s12935-022-02800-x (PMC9741792; doi:10.1186/s12935-022-02800-x)
Supplement: Supplementary file 3 — Additional file 3: Supplementary Tables. Table S1. Primer sequences for genotyping. [file 12935_2022_2800_MOESM3_ESM.docx]

**ADDITIONAL TABLES**

**Table S1 Primer sequences for genotyping**

| **Primers for genotyping** | **Sequence** |
| --- | --- |
| LYVE1_KO_CM3145F (Forward) | AGCTGGGAACAAAGCTGGAAGCAA |
| LYVE1_KO_WT3513R (Reverse) | TTCCCACACCTGGGGTTTGAGAA |
| LYVE1_KO_KO6956R (Reverse) | ACTAGGGCTCACAACCCTCTTTA |
